# Mapping Collective Electron-Density Rearrangements at Oil–Water Interfaces Using Optimal Transport

**DOI:** 10.1021/acs.jctc.6c01556

**Published:** 2026-10-21

**Authors:** Gabriele Amante, Klaudia Mráziková, Gabriele Centi, Sylvie Roke, Ali Hassanali, Giuseppe Cassone

**Affiliations:** † Institute for Chemical-Physical Processes, 9327 National Research Council (CNR-IPCF), V.le F. Stagno d’Alcontres 37, Messina 98158, Italy; ‡ Department of Chemical, Biological, Pharmaceutical, and Environmental Sciences, 18980 University of Messina, Viale F. Stagno d’Alcontres 31, Messina 98166, Italy; § J. Heyrovský Institute of Physical Chemistry, Academy of Sciences of the Czech Republic, Dolejškova 3, Prague 8 CZ-18223, Czech Republic; ∥ Laboratory for Fundamental BioPhotonics (LBP), Institute of Bioengineering, 27218 École Polytechnique Fédérale de Lausanne (EPFL), Lausanne 1015, Switzerland; ⊥ 18473 International Centre for Theoretical Physics (ICTP), Strada Costiera 11, Trieste 34151, Italy

## Abstract

The electronic and spectroscopic properties of aqueous hydrophobic interfaces continue to be a topic of active research. One such open question is rooted in the origin of the apparent negative charge of oil droplets. Here, we deploy an information-theoretic optimal transport approach dubbed VIPER-CT (Voronoi-Informed Partitioning of Electron Redistribution for Charge Transfer), to construct the electronic *T*-matrix derived from the real-space electron density determined from first-principles simulations of an extended decane-water interface, sampled using neural-network-based Deep Potential Molecular Dynamics. This matrix operationally separates directional interphase charge transfer (CT) from intraphase density rearrangements. While decane-water molecular clusters of various sizes show small net CT from decane to water, the extended interface displays a systematic opposite electronic asymmetry, yielding a net CT from water to the hydrocarbon phase producing an average surface charge density of ∼0.003 *e* ^–^ nm^–2^ on the oil phase. This imbalance is accompanied by much larger intraphase self-polarization, particularly within the hydrocarbon phase, demonstrating that collective many-body polarization dominates the interfacial electronic response.

## Graphical abstract

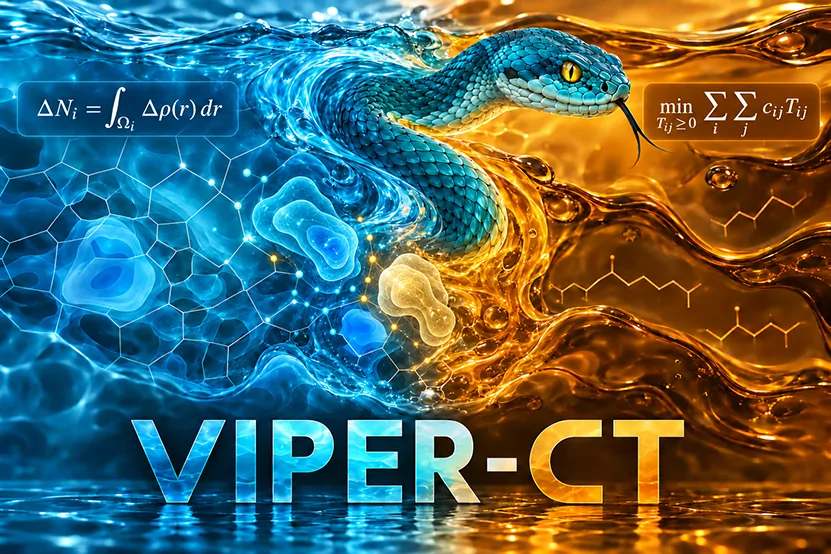

## Introduction

Aqueous hydrophobic interfaces are ubiquitous in physics, chemistry and engineering applications. Within this context, oil–water interfaces remain one of the most well studied playing a central role in a wide range of phenomena spanning soft-matter physics,^1,2^ colloid science,^3,4^ atmospheric chemistry,^5,6^ and microdroplet reactivity.^7–10^ A particularly intriguing and long-standing observation is that oil droplets dispersed in water acquire a sizable negative ζ-potential even in the absence of added surfactants or electrolytes. This charge stabilizes emulsions against coalescence^11^ and drives the electrophoretic mobility of oil droplets in the presence of external electric fields.^12^ Recent spectroscopic measurements inferred the existence of exceedingly large interfacial electric fields (∼50–90 MV cm^–1^) associated with the droplet ζ-potential which have been proposed to be important for enhanced reactivity at interfaces.^13^ Because the ζ-potential exhibits a clear pH dependence, the negative charge has traditionally been attributed to hydroxide ions (OH^–^) adsorbing at the hydrophobic interface.

Direct experimental and theoretical evidence for hydroxide accumulation at such interfaces has remained elusive below bulk concentrations of ∼1 M (pH 14). Several label-free, surface-specific spectroscopic methods designed to probe hydroxide directly—such as charge-transfer-to-solvent (CTTS) transitions,^14^ 2p-π electronic transitions,^15^ and the O–H^–^ vibrational stretch^16–21^ – do not reveal any pH-dependent interfacial accumulation of OH^–^ below pH ∼13–14 (corresponding to ∼100 mM–1 M). Similarly, most molecular simulations suggest that hydroxide ions are repelled from hydrophobic surfaces or at best, too weakly attracted to account for the large negative zeta potentials.^12,22–25^ This has thus motivated the search for alternative explanations for the origin of the interfacial charge.

One frequently invoked mechanism attributes the negative charge of oil droplets to charge transfer (CT) at the oil–water interface. Evidence for this mechanism has primarily been motivated from electronic-structure calculations of the water dimer^26^ combined with molecular dynamics simulations of extended interfaces, which reveal pronounced hydrogen-bond asymmetries that are typically enhanced at water surfaces.^20,27,28^ While early studies focused on CT between water molecules, Poli and coworkers proposed that oriented interfacial water molecules could donate electronic charge to hydrocarbon groups,^28^ thereby generating a negatively charged oil phase. Subsequent surface-sensitive vibrational spectroscopy measurements revealed distinct interfacial signatures in both the O–H and C–H stretching regions. These features were interpreted as evidence for *improper* C–H···O hydrogen bonds that could facilitate CT from water to the hydrocarbon phase.^11^

The magnitude and spatial extent of CT, however, depend sensitively on both the level of electronic-structure theory and the scheme used to partition the electron density.^28^ More fundamentally, CT is not itself a quantum-mechanical observable, and its numerical magnitude necessarily depends on the fragmentation, reference electronic state, and real- or Hilbert-space representation adopted. This ambiguity has accompanied interaction analyses since the pioneering Kitaura-Morokuma variational decomposition of the Hartree–Fock interaction energy into electrostatic, exchange-repulsion, polarization, CT, and residual mixing contributions,^29^ and remains central to modern comparisons between real-space charge-displacement and orbital-based ALMO analyses.^30^ In this context, recent energy-decomposition analyses of water-hydrocarbon molecular clusters have argued that forward (water → oil) and backward (oil → water) CT contributions largely cancel, resulting in negligible net CT and attributing spectroscopic shifts primarily to Pauli repulsion and polarization effects.^31^ While such cluster-based approaches provide valuable insight into individual intermolecular interactions, they are intrinsically limited in their ability to capture the collective, many-body, and nonlocal polarization effects that emerge only at extended interfaces.

Here, we revisit the molecular and electronic origins of CT at extended oil–water interfaces by combining Deep Potential Molecular Dynamics simulations of a decane-water interface with first-principles electronic-structure calculations. By explicitly probing how the real-space electron density responds to interface formation using an information-theoretic analysis borrowing ideas from optimal transport theory, we develop the VIPER-CT (Voronoi-Informed Partitioning of Electron Redistribution for Charge Transfer) scheme and obtain an atom-resolved picture of interaction-induced charge rearrangements. The VIPER-CT framework allows us to disentangle interphase CT from intraphase polarization and directly quantify both forward and backward CT processes at the interface. Our results reveal that a net transfer of electronic charge from water to the oil phase emerges only at the extended interface and is not captured by isolated molecular cluster models. The resulting charge redistribution is accompanied by structural distortions of the interfacial hydrocarbon groups that are consistent with the experimentally observed blue shift of interfacial C–H vibrational modes, providing an unprecedented direct molecular-level picture of interaction-induced charge rearrangements at oil–water interfaces.

## Methods

### Deep Potential Molecular Dynamics and Electronic Structure Calculations

With the aim of exploring the configurational space accessible to an extended oil–water interface, a Deep Potential Molecular Dynamics (DPMD) simulation of a decane-water slab was carried out with the LAMMPS software (v. 29Aug2024).^32^ The reactive DP reported in ref 25, which was trained to reproduce DeePKS-generated reference data with approximate M06–2X^33^ accuracy, was employed. The simulation box was composed of 15 decane (C_10_H_22_) and 156 water (H_2_O) molecules (i.e., 948 atoms) arranged in a parallelepiped box of *a* = *b* = 19.641 Å and *c* = 25.000 Å yielding an average density of 0.73 and 1.00 g·cm^–3^ in the decane and water subsystems, respectively, consistent with previous studies.^28,34^ The DPMD simulation was carried out under periodic boundary conditions and in the NVT canonical ensemble for 5 ns. The first ns was employed for equilibration, the last 4 ns were used for analyses. The temperature was kept fixed at 300 K via the canonical-sampling-velocity-rescaling (CSVR) thermostat^35^ with a damping factor of 0.04 ps.

1000 randomly chosen configurations were extracted from the DPMD trajectory and used as input for *ab initio* calculations at the r2SCAN^36^ Density Functional Theory (DFT) level. In addition to a series of DFT benchmarks performed on several dimer species and up to the coupled-cluster singles and doubles (CCSD) theory,^37–40^ preliminary tests on 100 extended decane-water configurations extracted from the DPMD simulations were carried out at the BLYP+D3,^41–44^ SCAN,^45^ and r2SCAN^36^ levels to compare the current results with previous GGA-based evaluations^28^ and to understand the impact of the electron delocalization in charge transfer (CT) quantification, as reported in the Supporting Information (SI). Calculations on molecular clusters of various sizes were performed via the Orca software (v. 6.1)^46^ in the gas-phase, while all periodic electronic structure calculations of the extended oil–water interface were carried out by employing the CP2K software (v. 2024.3).^47^ Wave functions of the atomic species have been expanded in TZVP basis sets with Goedecker-Teter-Hutter (GTH) pseudopotentials using the GPW method.^48^ In addition, a large plane-wave cutoff of 1200 Ry has been imposed, as typically required for r2SCAN calculations.

To test explicitly whether the CT asymmetry emerges already in finite, increasingly microsolvated clusters, we additionally extracted decane-water clusters containing one, two, or three decane molecules and systematically varied the number of surrounding water molecules. For one decane, ensembles of 100 configurations were analyzed for hydration numbers $N_{H_{2}O}=2,4,6,\;\mathrm{and}\;8$ ; for two and three decanes, 50 configurations were analyzed at each $N_{H_{2}O}=2,4,6,8,10,\;\mathrm{and}\;12$ . The same frozen-fragment density-difference and optimal-transport protocol was applied to these clusters.

### Charge Transfer Analysis

A simple approach to quantify CT was proposed by Belpassi and coworkers,^49,50^ based on directly comparing the electron density of a complex with that of its isolated fragments. This method accurately captures charge displacements in weakly interacting systems, such as noble gases interacting with hydrating water, in agreement with molecular beam scattering measurements probing interatomic potentials.^51–53^ As shown in the SI, our VIPER-CT (Voronoi-Informed Partitioning of Electron Redistribution for Charge Transfer) approach yields excellent agreement with the Belpassi scheme for CT in the water dimer.^50^

In the VIPER-CT method, we quantify interfacial CT from first-principles electron densities. Let ρ_*AB*_(**r**) be the electron density of the full oil–water interface in the analyzed geometry, and let $\rho_{A}^{\mathrm{iso}}\left(r\right)$ and $\rho_{B}^{\mathrm{iso}}\left(r\right)$ be the densities of the two isolated subsystems computed in the same frozen nuclear geometry as in the complex and within the same simulation box. The interaction-induced density rearrangement is

1 $$\Delta \rho \left(r\right)=\rho_{AB}\left(r\right)-\left[\rho_{A}^{\mathrm{iso}}\left(r\right)+\rho_{B}^{\mathrm{iso}}\left(r\right)\right]$$

which by construction conserves the number of electrons,

2 ∫Δρ(r)dr=0

We partition the space into atom-centered cells {Ω_*i*_} using a van der Waals- (vdW-)­weighted power diagram to account for different atomic sizes (see SI for further information): for atom *i* at **R**_*i*_ with vdW radius $R_{i}^{\mathrm{vdW}}$ , we define the power distance *D*_*i*_(**r**) = ∥**r** – **R**_*i*_∥^2^ – *w*_*i*_ with weight $w_{i}={\left(R_{i}^{\mathrm{vdW}}\right)}^{2}$ , and set Ω_*i*_ = {**r** | *D*_*i*_(**r**) ≤ *D*_*j*_(**r**), ∀*j*}, where *j* runs over all atoms of the system. On the real-space grid (voxel volume *V*_cell_), we then form for each atom *i* the net electron change and its positive/negative parts:

3 $$\Delta N_{i}\equiv \int_{\Omega_{i}}\Delta \rho \left(r\right)dr\approx \underset{r_{g}\in \Omega_{i}}{\sum }\Delta \rho \left(r_{g}\right)V_{\mathrm{cell}}$$

4 $$A_{i}^{+}\equiv \int_{\Omega_{i}}\max\left(\Delta \rho \left(r\right),0\right)dr,,A_{i}^{-}\equiv \int_{\Omega_{i}}\max\left(-\Delta \rho \left(r\right),0\right)dr$$

so that $\Delta N_{i}=A_{i}^{+}-A_{i}^{-}$ , $A_{i}^{\pm }\geq 0$ , and $\underset{i}{\sum }A_{i}^{+}=\underset{i}{\sum }A_{i}^{-}$ (global conservation, eq 2), where *A*_*i*_ terms are the electron density accumulations/depletions. We report *q*_*i*_ ≡ Δ*N*_*i*_ in units of electrons (*e* ^–^): *positive q*_*i*_ means electron *accumulation* on atom *i* (i.e., a more *negative* physical charge −*e q*_*i*_); *negative q*_*i*_ means electron *depletion*. In practice, total charge conservation is satisfied to within a small numerical residual, with |∑_*i*_ Δ*N*_*i*_| and $\left|\underset{i}{\sum }A_{i}^{+}-\underset{i}{\sum }A_{i}^{-}\right|\approx 5\times 10^{-5}e^{-}$ , which we regard as the numerical error on the here provided CT measures.

To map where depleted electrons plausibly flow, we solve a size- and geometry-aware optimal transport problem over atoms, in the spirit of the Monge–Kantorovich optimal transport formulation.^54,55^ Let $D=\left\{i:A_{i}^{-}>0\right\}$ (donors/sources) with supplies $s_{i}=A_{i}^{-}$ , and $A=\left\{j:A_{j}^{+}>0\right\}$ (acceptors/sinks) with demands $d_{j}=A_{j}^{+}$ . We seek a non-negative *T*-matrix $T=[T_{ij}{]}_{i\in D,j\in A}$ in *e* ^–^ such that

5 $$\underset{j\in A}{\sum }T_{ij}=s_{i},\left(\forall i\in D\right),,\underset{i\in D}{\sum }T_{ij}=d_{j}\left(\forall j\in A\right)$$

and which minimizes the total pairing cost.

6 $$\underset{T_{ij}\geq 0}{\min}\underset{i\in D}{\sum }\underset{j\in A}{\sum }c_{ij}T_{ij}$$

Distances *d*_*ij*_ = ∥**R**_*j*_ – **R**_*i*_∥ are computed with the minimum-image convention consistent with the simulation cell; we use a vdW-normalized gap

$$c_{ij}=\frac{d_{ij}}{R_{i}^{\mathrm{vdW}}+R_{j}^{\mathrm{vdW}}},,c_{ij}\leftarrow M\;\mathrm{if}\;d_{ij}>\mathrm{cutoff}$$ 7

where a large *M* enforces a soft distance cutoff. Small imbalances between ∑_*i*_ *s*_*i*_ and ∑_*j*_ *d*_*j*_ due to discretization are removed by rescaling the larger total to the smaller before solving eq 6. The solution *T* is elementwise non-negative and represents the *number of electrons* reassigned from each donor atom to each acceptor atom under the chosen geometric cost. Forward/backward and net interphase CT then follow directly by summing up all *T*_*ij*_ across phases. With H (hydrocarbon atoms) and W (water atoms),

8 $${\mathrm{CT}}_{W\to H}^{\left(T\right)}=\underset{i\in W\cap D}{\sum }\underset{j\in H\cap A}{\sum }T_{ij},,{\mathrm{CT}}_{H\to W}^{\left(T\right)}=\underset{i\in H\cap D}{\sum }\underset{j\in W\cap A}{\sum }T_{ij}$$

9 $${\mathrm{CT}}_{\mathrm{net}}^{\left(T\right)}={\mathrm{CT}}_{W\to H}^{\left(T\right)}-{\mathrm{CT}}_{H\to W}^{\left(T\right)}=\underset{i\in H}{\sum }\Delta N_{i}=-\underset{i\in W}{\sum }\Delta N_{i}$$

so the transport-based net CT is numerically consistent with the density-partition result, up to the same ∼5 × 10^–5^ *e* ^–^ conservation residual. The vdW-weighted Voronoi partitioning included in our VIPER-CT approach applied to the real-space density difference Δρ­(**r**) enables a physically consistent assignment of electrons to atoms (Table S1) and quantification of interaction-induced charge rearrangements (Table S2). These contributions are summarized in a compact 2 × 2 electronic *T*-matrix notation:

10 $$T^{\left(2\times 2\right)}=\left(\begin{array}{ll} T_{W\to W} & T_{W\to H}\\ T_{H\to W} & T_{H\to H} \end{array}\right),,T_{X\to Y}=\underset{i\in X\cap D}{\sum }\underset{j\in Y\cap A}{\sum }T_{ij},,X,Y\in \left\{W,H\right\}$$

where diagonal elements describe intramolecular intraphase charge rearrangements and off-diagonal elements represent interphase charge-displacement contributions, which represent the main outputs of the VIPER-CT methodology here presented.

## Results and Discussion

### Charge Transfer in Dimers and Clusters

As a first validation of the VIPER-CT (Voronoi-Informed Partitioning of Electron Redistribution for Charge Transfer) framework, we apply the present methodology to a set of prototypical molecular dimers exhibiting different interaction strengths and bonding motifs. These simple systems provide a controlled benchmark to assess whether our method can discriminate between distinct interaction regimes—from strong hydrogen bonds to weak van der Waals contacts—while consistently quantifying both intermolecular charge transfer (CT) and intramolecular polarization. Shen and Head-Gordon^30^ have recently shown that the standard charge-displacement approach introduced by Belpassi and co-workers^49,50^ combines Pauli-driven deformation, polarization, and CT contributions, while relying on the delicate identification of an isodensity point. While we acknowledge these limitations—which reflect the intrinsically nonunique and reference-dependent nature of CT—our vdW-Voronoi optimal-transport VIPER-CT scheme addresses this main practical issue by avoiding any molecular-axis or isodensity-boundary definition and mapping the density rearrangement into directional atom- and phase-resolved contributions. The resulting *T*-matrix should therefore be regarded as an operational real-space descriptor, without requiring its individual elements to reproduce ALMO-CTA populations quantitatively. Unlike Hilbert-space methods such as ALMO-CTA,^56^ which require an orbital representation and become computationally demanding for extended systems, the real-space partitioning of VIPER-CT is directly applicable to periodic interfaces. The 2 × 2 *T*-matrices reported in Figure 1 exemplify this capability, providing a compact descriptor of intra- and intermolecular electronic rearrangements.

**Figure 1:**
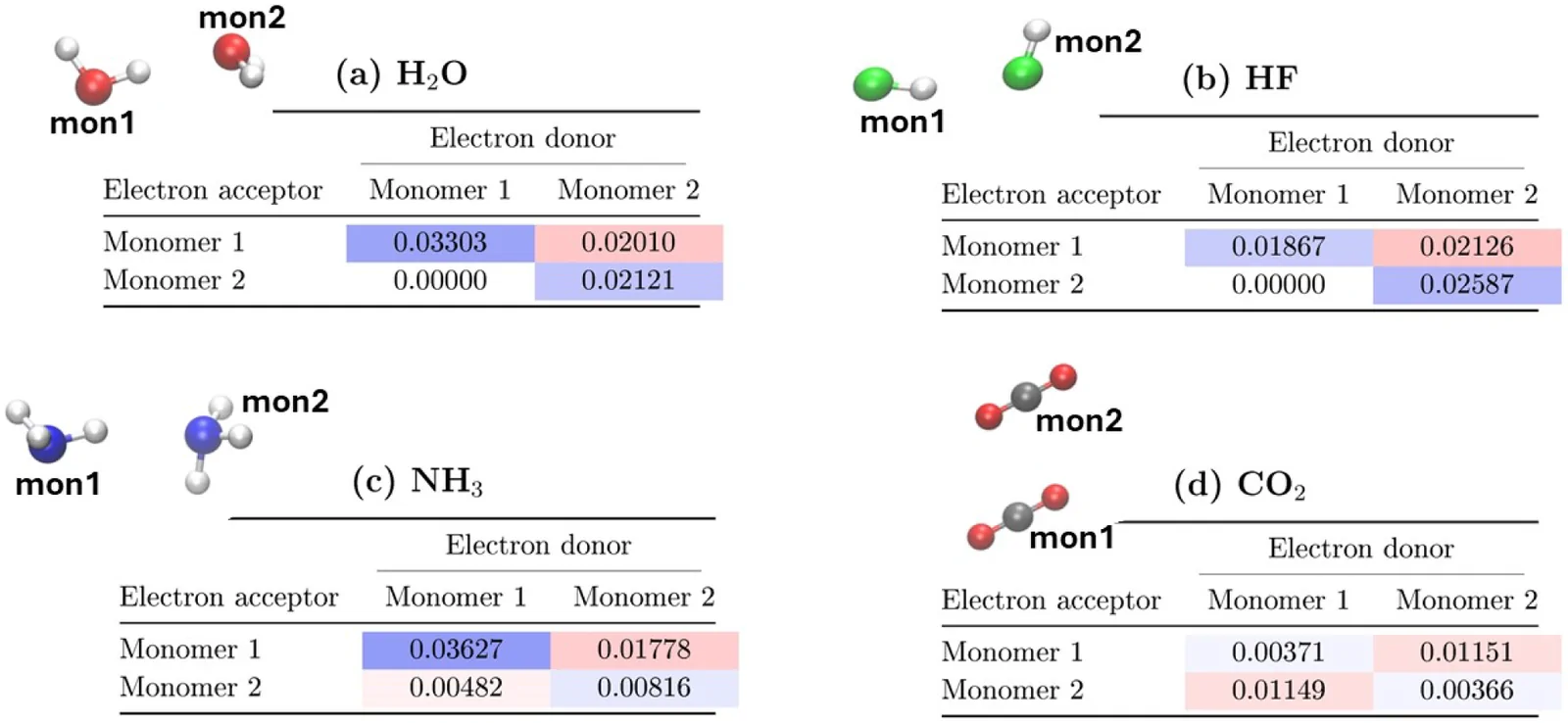
Electronic *T*-matrix of the water (a), hydrogen fluoride (b), ammonia (c), and carbon dioxide (d) dimer. Molecular geometries are optimized at the M06–2X/def2-TZVPD level whereas the respective electron densities are evaluated at the r2SCAN/def2-TZVPD theory. Diagonal elements (blue) correspond to self-polarization while off-diagonal entries (red) correspond to the intermolecular charge transfer. Net charge transfer is simply obtained by taking the absolute difference between off-diagonal elements. Color intensity of the cells follows the magnitude of the elements over the four *T*-matrices of the figure while zero values are not colored.

Strong hydrogen bonding in the water (Figure 1a) and hydrogen fluoride (Figure 1b) dimers produces a characteristic *T*-matrix pattern where one off-diagonal element reaches ∼0.020 *e* ^–^ (20 *me* ^–^) while the other is essentially zero. As discussed in the SI, this asymmetry reflects partial electron transfer from the hydrogen-bond acceptor (monomer 2) to the donor (monomer 1).^26,57^ The diagonal self-polarization terms (blue) show opposite trends in the two systems: in the water dimer the donor is more strongly repolarized, whereas in HF the acceptor experiences a larger polarization. This difference arises from the interplay between molecular polarizability, bond polarity, and geometry: the nearly orthogonal hydrogen-bond arrangement in the planar HF dimer enhances polarization of the acceptor lone-pair region, while the more cooperative orbital alignment in the water dimer, favors stronger repolarization of the donor unit.

The ammonia dimer is more subtle because its potential energy surface is rather flat, leading to highly floppy structures around the stationary points.^58^ We therefore analyze the linear staggered structure with C_*s*_ symmetry. The hydrogen-bond signature clearly appears in the corresponding *T*-matrix (Figure 1c), although the net CT (CT_*net*_ = |*T*_*mon*2→*mon*1_ – *T*_*mon*1→*mon*2_| ≈ 0.013 *e* ^–^) is smaller than in the H_2_O and HF dimers, indicating a less efficient hydrogen bond for CT. The NH_3_ dimer also exhibits asymmetric self-polarization, with the donor fragment undergoing stronger repolarization (T_*mon*1→*mon*1_) than the acceptor (T_*mon*2→*mon*2_). A markedly different pattern arises for the CO_2_ dimer (Figure 1d). Owing to its weak van der Waals interaction and centrosymmetric parallel-slipped C_2*h*_ structure, the system shows very small and nearly symmetric repolarization effects. Despite finite CT contributions on each monomer, the net CT essentially vanishes (CT_*net*_ ∼ 2 × 10^–5^ *e* ^–^).

One of the main goals of this work is to examine the CT between decane and water specifically comparing how this effect changes moving from isolated clusters to a realistic extended interface. We first begin by exploring the CT we extract for the decane-water dimers in vacuum. Following a similar strategy to that adopted in ref 31, we have extracted 1500 local configurations of decane-water dimers from our Deep Potential Molecular Dynamics (DPMD) trajectory of the extended decane-water interface, as visualized in Figure 2a. We focused on two types of typical configurations found at the oil–water interface as depicted in the yellow and green images of Figure 2a, corresponding to situations where the water hydrogens are either oriented toward or away from the decane molecule, respectively. Only pairs involving atoms classified as interfacial through the instantaneous Willard-Chandler (WC) surface^59^ were considered, using the thresholds *d*_C_ = 2.8 Å and *d*_W_ = 3.0 Å chosen on the basis of the respective location of the minimum along the density profile.

**Figure 2:**
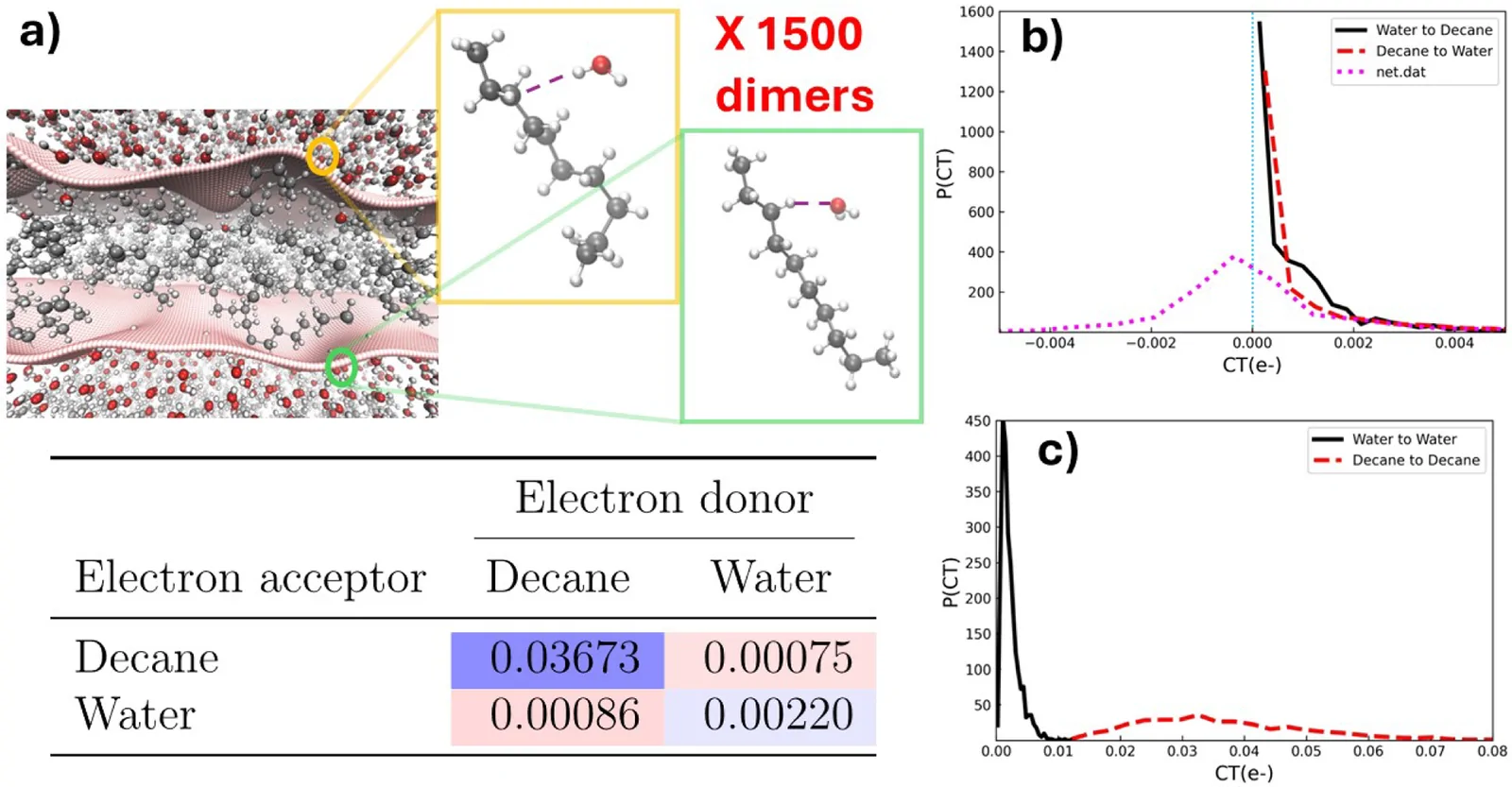
(a) Snapshot taken from the Deep Potential Molecular Dynamics simulation of the extended decane-water interface and the extraction procedure of decane-water dimers along with the respective *T*-matrix averaged over 1500 configurations. (b) Distributions of the charge transfer (CT, in *e* ^–^) by direction. Black solid line: water → decane CT; red dashed line: decane → water CT; magenta dotted line: net CT. (c) Distributions of the intramonomer (self-polarization) CT. Black solid line: water → water; red dashed line: decane → decane.

Accumulating all the per-atom charge fluctuations from the atoms involved in this dimer configuration, we observed that the self-polarization associated with decane is markedly larger than the water one, as quantified by the diagonal terms of the *T*-matrix reported in Figure 2a and by the probability distributions of Figure 2c. More importantly, an almost symmetric CT between decane and water emerges from this analysis. In fact, the absolute difference of the off-diagonal elements of the *T*-matrix produces an insignificant CT of 0.00011 *e* ^–^ (0.11 *me* ^–^) imbalance toward water, as quantified in Figure 2a and visualized in Figure 2b by the net CT curve (dotted magenta). This almost-zero net CT between hydrocarbon and water dimers is consistent with recent ALMO-EDA^60^ simulations recently reported by the Head–Gordon group^31^ on hexane-water systems. These findings imply that the CT associated with decane-water dimers are essentially insignificant.

The dimer result raises an important question: does the asymmetry of the extended interface appear gradually once several waters and/or hydrocarbons are included, or does it require a genuinely extended interfacial environment? We therefore repeated the VIPER-CT analysis for systematically microsolvated clusters containing one to three decane molecules and increasing hydration numbers (see Methods and SI). As reported in Figure 3, in all finite clusters examined, the net imbalance remains small and points from decane toward water, i.e., opposite to the water → decane imbalance of the extended periodic slab. Averaged over hydration number, the magnitude of this cluster imbalance is approximately 0.00219 *e* ^–^ (2.19 *me* ^–^) for one decane, 0.00387 *e* ^–^ (3.87 *me* ^–^) for two decanes, and 0.00394 *e* ^–^ (3.94 *me* ^–^) for three decanes. Increasing hydration therefore enhances the total intracluster electronic reorganization but does not induce a crossover to the water → oil direction observed in the extended slab. These tests show that the local forward/backward contact geometry is not by itself sufficient to explain the sign of the extended-interface CT.

**Figure 3:**
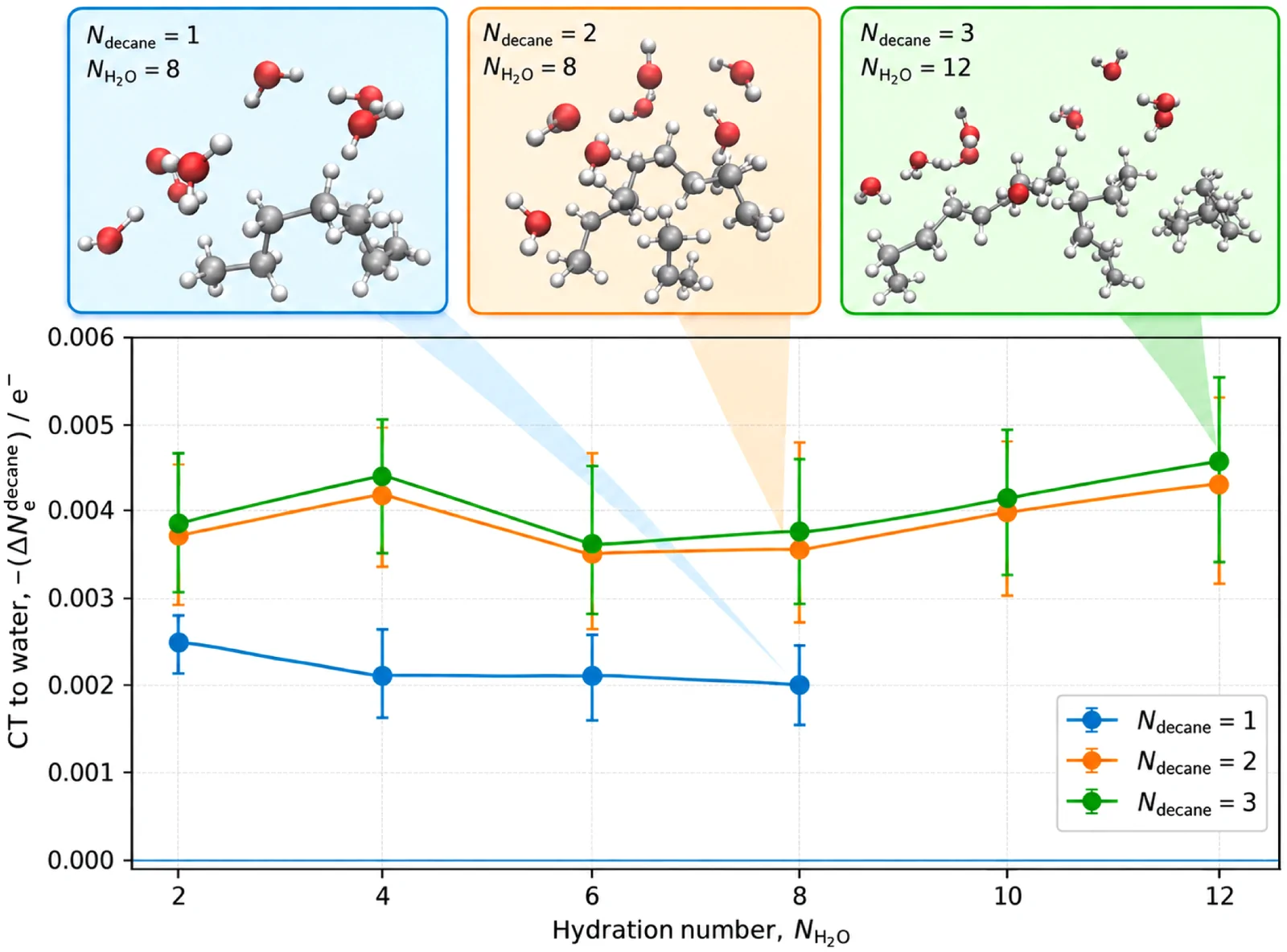
Net charge transfer (CT) from decane to water as a function of hydration number for clusters containing one, two, and three decane molecules. Positive values denote a net decane → water electronic imbalance. Error bars represent the standard deviation over the independently sampled cluster ensembles. Continuous horizontal light blue line marks the zero net charge transfer. On the top, representative structures of the molecular clusters are displayed.

Furthermore, Figure 3 emphasizes two complementary trends. First, increasing the number of decanes raises the magnitude of the small reverse cluster imbalance from approximately 2 *me* ^–^ for one decane to approximately 4 *me* ^–^ for two and three decanes, while hydration itself produces only modest, nonmonotonic variations. Second, the detailed *T*-matrices reported in Figures S1–S3 of the SI show that the diagonal self-polarization terms increase strongly with cluster size and hydration whereas the off-diagonal terms remain much smaller. Thus, making the local environment larger clearly increases the amount of electronic reorganization, but it does not drive that reorganization into the water → decane direction characteristic of the extended slab (see below). This distinction between growing polarization and the absence of a CT sign crossover is the key evidence that the periodic-interface bias is not a simple sum of isolated pairwise contacts, an aspect investigated in the next section.

### Charge Transfer at the Extended Decane-Water Interface

We begin by quantifying the electronic response that develops upon formation of the decane-water extended interface. Using the VIPER-CT methodology based on the electron density decomposition and CT evaluation introduced above, we (i) resolve the atom-specific net charge response as a function of the cross-phase distances, (ii) determine the net CT direction and the amount of the interfacial CT between the two phases, and (iii) isolate the intraphase self-polarization occurring independently within water and within the hydrocarbon matrix. Figure 4 summarizes these three complementary results.

**Figure 4:**
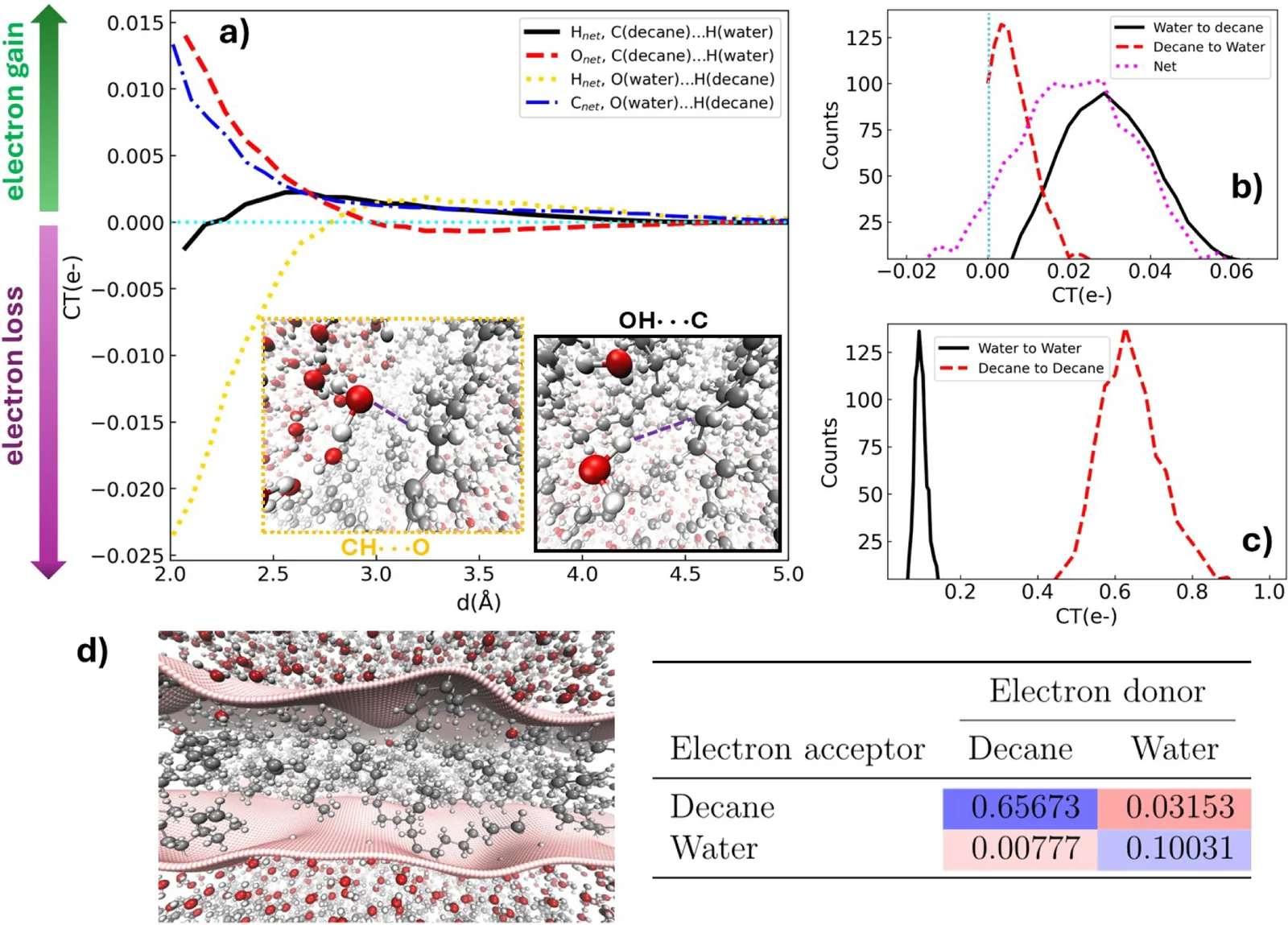
(a) Conditional mean of the net atomic charge, ⟨*q* ^net^⟩ (in *e* ^–^), plotted as a function of the cross-phase approach distances. Curves: black solid, water H net charge; red dashed, water O net charge; orange dotted, decane H net charge; blue dash-dot, decane C net charge. The thin cyan dotted horizontal line denotes *q* ^net^ = 0. Values are shown as bin-averaged means of ⟨*q* ^net^⟩ conditioned on the distance of the interaction. Yellow dotted inset displays the forward interaction (decane to water). Black continuous inset shows the backward interaction (water to decane). (b) Distributions of interfacial charge transfer (CT, in *e* ^–^) by direction. Black solid line: water → decane CT; red dashed line: decane → water CT; magenta dotted line: net CT. (c) Distributions of intraphase (self-polarization) CT. Black solid line: water → water; red dashed line: decane → decane. (d) Snapshot from a Deep Potential Molecular Dynamics trajectory of the decane-water slab, showing the Willard-Chandler instantaneous interface between the two phases (pale pink dots). Carbon, oxygen, and hydrogen atoms are depicted as silver, red, and white spheres, respectively. On the right, the respective average coarse-grained 2 × 2 *T*-matrix with the electron density resampled @r2SCAN/TZVP level. Entries are in *e* ^–^.

Figure 4a reports the conditional mean of the net atomic charge lying on the atomic species as a function of the cross-phase approach distance *d*, providing an atom-resolved picture of the different interfacial CT terms. In particular, the four CT curves for the water hydrogens (black solid), the water oxygens (red dashed), the decane hydrogens (yellow dotted), and the decane carbons (blue dash-dotted) are plotted as a function of two different relative distances: the C_(*decane*)_–H_(*water*)_ (black solid and red dashed lines) and the O_(*water*)_–H_(*decane*)_ (yellow dotted and blue dash-dotted lines). As expected, all curves converge toward zero at larger, bulk-like separations, confirming that the charge response induced upon interface formation is relatively short-ranged along directions approximately normal to the interface itself.

Within the water phase along the C_(*decane*)_ ··· H_(*water*)_ distance ranging between ∼2–3 Å, oxygen atoms (red dashed curve) at contact exhibit a partial electron accumulation due to the hydrogen bonds they donate to decane (O–H···C), while at farther distances, the observed electron density depletion mirrors their involvement as hydrogen-bond acceptors in C–H···O interactions. Water hydrogens display the opposite response (solid black curve), exhibiting charge depletion at the topmost interfacial water layer (∼2.0–2.2 Å), where some of the hydrogen atoms of the water subsystem are capable of donating efficient forward hydrogen bonds to the decane moiety. However, the remainder water’s hydrogen atoms accumulate some extra electron charge within a relatively large range of distances of interactions established with the oil phase (∼2.3–4.1 Å). The sign of this CT arises from a mixture of cooperative effects including the response from waters losing electron density in C–H···O interactions as well as hydrogens in dangling (free O–H’s) at the interface.

At close contact with water, carbon atoms on the hydrocarbon side acquire a negative mean charge, indicating electron accumulation on interfacial carbons. Although the short-range CT behavior resembles that of water oxygen atoms, decane carbons remain more negatively charged than their bulk counterparts over the entire interaction range up to ∼5.0 Å, as indicated by the positive blue dash-dotted curve in Figure 4a. Conversely, the hydrocarbon’s hydrogen atoms (yellow dotted line) become electron-poor when directly engaged in cross-phase contacts up to a O_(*water*)_ ··· H_(*decane*)_ distance of ∼2.8 Å, where the respective CT curve crosses zero and changes sign. This indicates that whereas the decane’s hydrogen atoms donating hydrogen bonds to water get positively charged, their counterpart lying on the opposite side of the hydrocarbon backbone tends to acquire a partially negative charge, similarly to their covalently bound carbon atoms. Notably, the spatial extent of this partially charged region is larger in the oil phase than in the water, as witnessed by the nonzero values of the H_*net*_ and C_*net*_ curves up to ∼5.0 Å of the O_(*water*)_ ··· H_(*decane*)_ distance, while the H_*net*_ and O_*net*_ CT curves referred to the aqueous subsystem approach the bulk-like zero net CT already at about 4.0 Å of the C_(*decane*)_ ··· H_(*water*)_ distance.

The directionality of the interfacial charge flow is illustrated in Figure 4b, which reports the distributions of the CT between each subsystem. By construction, the charge donated by each subsystem is a positively defined quantity. The amount of CT transferred from decane to water (red dashed curve) attains a maximum value of ∼0.020 *e* ^–^ (20 *me* ^–^). In contrast, the forward CT distribution from water to decane (black solid curve) is shifted toward larger values than the backward CT from decane to water, resulting in a positive net CT with an average of 0.024 *e* ^–^ (24 *me* ^–^) and fluctuations reaching up to 0.060 *e* ^–^ (60 *me* ^–^). These values are quantified in the average 2 × 2 *T*-matrix reported in Figure 4d, where a net average CT of 0.02376 *e* ^–^ from water to decane emerges. This imbalance indicates *an overall accumulation of electronic charge on the hydrocarbon side of the interface* induced by the interaction with water, a feature that is not captured when the analysis is conducted on water-decane molecular clusters (Figures 2 and 3). As an independent periodic benchmark, we also evaluated the density-derived atomic point charges (DDAPC),^61^ whose detailed analyses are reported in the SI (Figure S4). In a nutshell, the DDAPC partition independently assigns a negative average partial charge to the hydrocarbon phase and a positive charge of equal magnitude to water, consistent in sign with the interfacial asymmetry obtained via the Δρ-based VIPER-CT scheme. Incidentally, this is further corroborated by another independent analysis of the Maximally Localized Wannier Function (MLWF)^62^ centers (Figure S5 of the SI). With decane removed, water molecules retaining interfacial geometries exhibit smaller dipole magnitudes than bulk-like molecules, revealing a structural polarization signature associated with their local hydrogen-bonding environment (Figure S5-a). Restoring decane at fixed water geometry produces a broad interfacial Δ|μ| distribution with positive and negative tails, whereas the bulk distribution remains narrowly centered near zero (Figure S5-b). The oil environment therefore induces a heterogeneous molecular response rather than a uniform dipole shift. Consistent with the analysis of Figure 4, these results support predominantly heterogeneous collective polarization, with a smaller systematic interphase CT bias superimposed.

Earlier we saw that for the decane-water clusters, a significant part of the electronic response involves the self-polarization contribution of the decane and water molecules. Figure 4c isolates the intraphase self-polarization induced upon interface formation. The hydrocarbon self-polarization distribution is centered at substantially larger values (∼0.6–0.7 *e* ^–^) than water (∼0.1–0.15 *e* ^–^), indicating that the decane phase accommodates the interfacial perturbation through a much stronger internal charge rearrangement, which is approximately 1 order of magnitude larger than that exhibited by water. This is of course rooted in the fact that the hydrocarbon is a larger molecule and therefore with more electronic degrees of freedom available to be perturbed by the creation of the interface.

Previous studies of CT mechanisms at air–water and oil–water interfaces suggest that interfacial charge accumulation stems from asymmetries in the populations of coordination defects with differing numbers of hydrogen-bond donors and acceptors.^20,28^ For the hexane-water interface, Zhao et al.^31^ showed that backward and forward CT balance each other leading to essentially no net charge displacement at the interface. To understand the origins of the CT at the decane-water interface, we examined the different possible orientations of water molecules relative to the instantaneous Willard-Chandler (WC) surface.^59^ Specifically, atoms are classified as interfacial when their distance from the WC surface satisfies |*d*_WC_(*x*, *y*, *z*) = *z* – *z*_WC_(*x*, *y*)| ≤ *d*_phase_, with *d*_C_ = 2.8 Å for decane carbons and *d*_W_ = 3.0 Å for water oxygens. This labeling allows us to distinguish two interaction motifs: the forward C–H···O and the backward O–H···C contacts, characterized by the corresponding approach distances and angles. Within our CT convention, the hydrogen-bond donor corresponds to the net electron-accepting side, so that the C–H···O motif correlates with CT from water to decane, and vice versa.

Figure 5 shows a clear asymmetry between the two cross-phase hydrogen-bond-like interactions. In Figure 5a, the backward O–H···C motif spans a broader region shifted toward longer distances and larger θ_HÔC_ angles, whereas the forward C–H···O motif (Figure 5b) samples shorter and more directional contacts. These geometries identify C–H···O contacts as a locally favorable channel for water-to-hydrocarbon density displacement. However, the microsolvation analysis reported in Figures 2 and 3 demonstrates that the same local geometric preference is not sufficient to determine the sign of the net CT: clusters containing up to three decanes and 12 waters still display a small net imbalance toward water. Thus, the contact asymmetry is a local structural ingredient of the electronic response, while the water-to-decane net CT bias is an emergent property of the extended interfacial organization and its many-body polarization environment, capable of accommodating an extended charge separation across different atomic layers, as shown in Figure 4.

**Figure 5:**
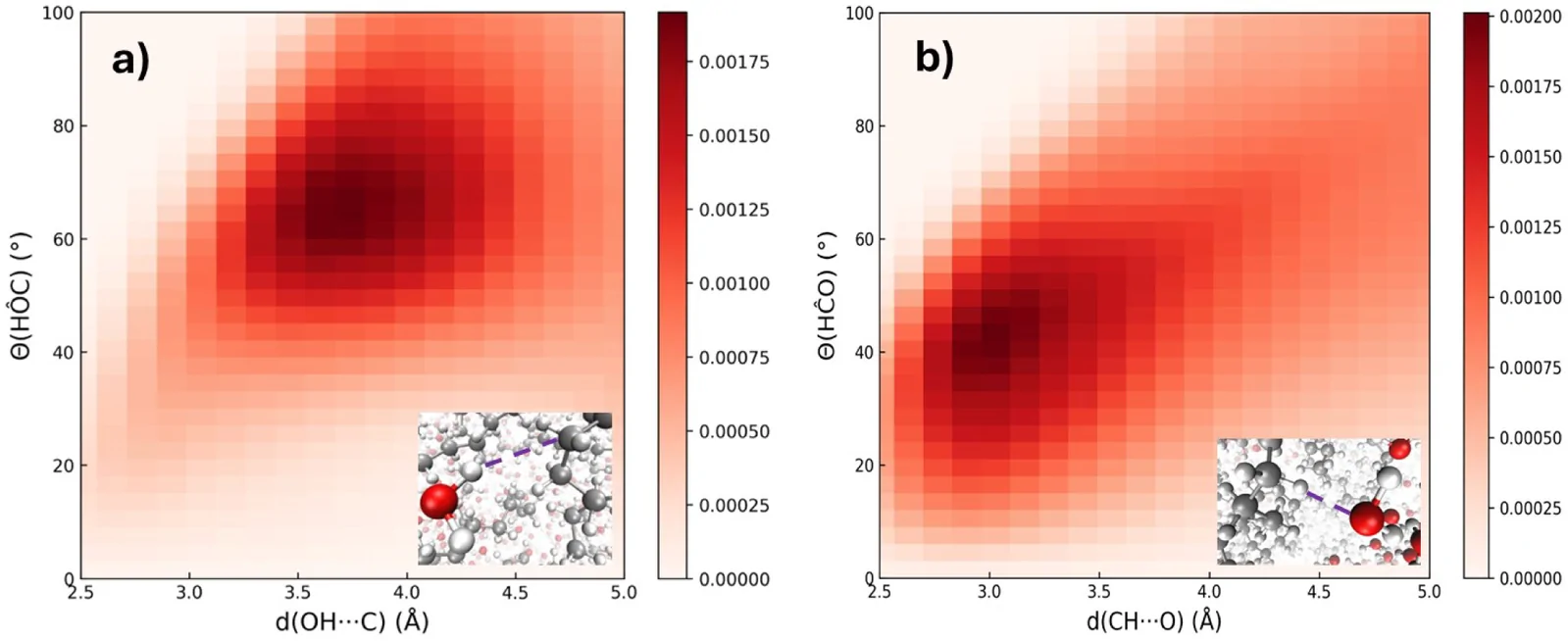
(a) Two-dimensional probability density *P*(*d*_OH···C_, θ_HOC_), where *d*_OH···C_ ≡ *d*(OH ··· C) and θ_HOC_ ≡ ∠(H_water_ – O – C) of the backward O–H···C interaction motif (water → decane). (b) Two-dimensional probability density *P*(*d*_CH···O_, θ_HCO_), where *d*_CH···O_ ≡ *d*(CH ··· O) and θ_HCO_ ≡ ∠(H_decane_ – C – O) of the forward C–H···O interaction motif (decane → water). Only atoms tagged as interfacial via the instantaneous Willard-Chandler surface are sampled, using thresholds *d*_C_ = 2.8 Å (carbons) and *d*_W_ = 3.0 Å (oxygens). Insets show representative configurations used to define the geometric descriptors.

### Structural Fingerprints of Charge Transfer at the Oil–Water Interface

The VIPER-CT inspection of the change in electron density underlying the creation of an extended decane-water interface reveals a collective process involving electronic polarization within the decane and water molecules as well as between the two phases. These mechanisms cannot be directly probed experimentally and are therefore typically inferred indirectly from surface sensitive vibrational spectroscopy^11,18,19^ which arise due to a geometrical response of the nuclear degrees of freedom. Since our CT analysis examines the electronic response at fixed nuclear geometry, we turn to examining structural properties of the two phases from the DPMD simulations which have effectively learned the electronic properties such as the energies and forces. Without explicitly computing relevant IR or Raman spectra, we can only make indirect suggestions on possible vibrational responses one might expect at the oil–water interface.

Figure 6 shows that the interface induces subtle but systematic and measurable modifications within both phases. On the water side (Figure 6a), the conditional mean ⟨*d*_OH,cov_ | *d*_WC_⟩—which represents the mean O–H covalent bond distance of the water molecules belonging to the interfacial region—is reduced at small distances from the WC interface, *d*_WC_, and gradually approaches the bulk mean upon moving away from the interface. This indicates a slight contraction of the O–H covalent bonds in the interfacial region. Concomitantly, the *d*_OO_ distribution monitoring the intermolecular distances between water molecules (Figure 6c) is shifted toward larger separations at the interface, consistent with a mild opening of the first coordination shell with respect to the typical bulk-like condition. Taken together, these structural changes would imply that the vibrational frequencies of the O–H oscillators at the interface would be slightly blue-shifted in frequency relative to those in the bulk. This likely originates from the presence of dangling O–H bonds from an enhanced concentration of hydrogen-bond defects.^28^

**Figure 6:**
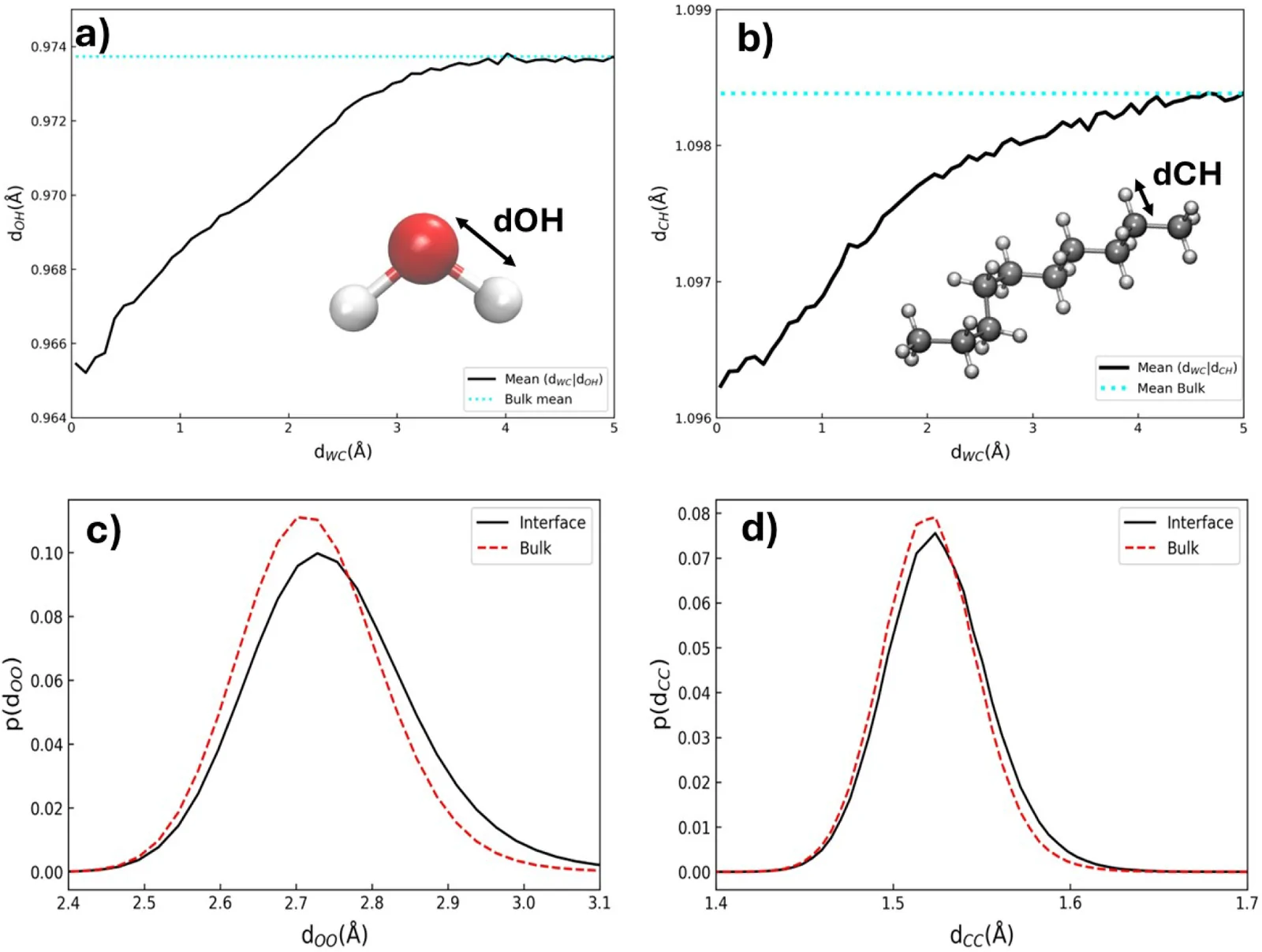
(a) Conditional mean of the covalent O–H bond length of water molecules, ⟨*d*_OH,cov_ | *d*_WC_⟩, as a function of the distance to the instantaneous WC surface, *d*_WC_ (black solid line). The cyan dotted line marks the bulk mean, ⟨*d*_OH,cov_⟩_bulk_. (b) Conditional mean of the covalent C–H bond length of decane molecules, ⟨*d*_CH,cov_ | *d*_WC_⟩, as a function of *d*_WC_ (black solid line), compared with the bulk mean, ⟨*d*_CH,cov_⟩_bulk_ (cyan dotted line). Note that the vertical scale in panel (b) is smaller than in panel (a), reflecting the reduced bond-length variations in decane. (c) Probability density of the nearest-neighbor O···O distance, *d*_OO_, in water: interface (black solid) vs bulk (red dashed). (d) Probability density of the nearest-neighbor C···C distance, *d*_CC_, in decane: interface (black solid) vs bulk (red dashed).

Structural changes in the water molecules do not directly probe the complex interactions at the interface. We thus turned next to examining the structural coordinates of the decane molecules. Although C–H bonds are less polarizable than O–H bonds, Figure 6b reveals a small decrease of the conditional mean ⟨*d*_CH,cov_ | *d*_WC_⟩—which represents the mean C–H covalent bond distance of the decane molecules belonging to the interfacial region—close to the WC surface, which relaxes toward the bulk value at larger *d*_WC_. Interestingly, this effect is consistent with experimental indications of interface-perturbed C–H bonds and the blue-shift of the respective stretching mode.^11^

To determine whether this contraction is specifically enriched among the C–H groups participating in the forward C–H···O motif, we further separated interfacial C–H bonds in hydrogen-bonded and non-hydrogen-bonded. C–H bonds assigned to the hydrogen-bond-like population account for 16.49% of the sampled interfacial C–H observations and have ⟨*r*_CH_⟩ = 1.0812 Å (standard deviation 0.0701 Å), compared with 1.0896 Å (standard deviation 0.0990 Å) for the non-hydrogen-bonded population. Thus, the C–H bonds directly engaged in C–H···O contacts are shorter on average by ∼0.0084 Å. Although the latter difference is tiny on an absolute bond-length scale, such a modest structural response is physically reasonable for a C–H covalent bond, whose electronic distribution and equilibrium bond length are comparatively weakly perturbed by the surrounding environment.

This structural correlation should not, however, be interpreted *tout-court* as demonstrating that CT is itself the origin of the blue shift. Adiabatic ALMO-EDA calculations on prototypical isolated blue-shifting C–H···Y complexes showed that Pauli repulsion is the only frozen-interaction component that contracts the C–H bond, whereas permanent electrostatics and dispersion favor bond elongation and CT produces a red-shifting contribution.^63^ These findings provide a valuable reference for individual intermolecular contacts, but their mechanistic decomposition cannot be assumed to remain unchanged at an extended and dynamically fluctuating oil–water interface, where collective many-body polarization, heterogeneous local fields, and the simultaneous action of multiple intermolecular contacts may alter the balance among the different contributions. The interfacial C–H contraction and net water-to-decane density displacement observed here should therefore be regarded as manifestations of this collective and complex electronic response, whose detailed decomposition cannot be inferred solely from isolated cluster models.

Similarly, although less markedly than the O···O distribution (Figure 6c), the nearest-neighbor C···C distribution (Figure 6d) is slightly shifted and broadened toward larger distances at the interface, indicating a modest loosening of the hydrocarbon backbone packing, consistent with the previously discussed accumulation of negative charge on interfacial carbon atoms. Notably, the fact that both *d*_OO_ and *d*_CC_ respond in the same direction—i.e., toward slightly larger first-neighbor separations —suggests that the interface promotes a modest local “opening” of the nearest-neighbor structure in each phase. Within this framework, the interface emerges as a region of enhanced structural and electronic susceptibility, where subtle but measurable net interphase charge displacement contributions results in nonlocal polarization and structural rearrangements. These collective effects appear to be at the basis of the observed vibrational shifts of specific modes at oil–water interfaces.

## Conclusions

In this work, we developed a real-space framework—dubbed as VIPER-CT, Voronoi-Informed Partitioning of Electron Redistribution for Charge Transfer—for quantifying interaction-induced charge rearrangements in extended molecular systems by integrating extensive neural-network-based Deep Potential Molecular Dynamics sampling with the *ab initio* evaluation of electron densities for representative interfacial configurations. Starting from the electron-density difference between the interacting system and its frozen isolated subsystems, the VIPER-CT method employs a van der Waals-weighted, atom-centered partitioning of real space. The resulting atomic electron accumulations and depletions are then coupled through a geometry-aware optimal-transport problem, translating the complex electronic response into a compact transport *T*-matrix that resolves directional interphase charge transfer (CT) and intraphase electronic rearrangements. By combining extensive configurational sampling with an atomically resolved description of electron-density redistribution, the VIPER-CT framework is naturally suited to periodic and dynamically fluctuating interfaces, where conventional orbital-based energy-decomposition approaches can become computationally prohibitive.

We validated the methodology on prototypical molecular clusters and subsequently applied it to the extended decane-water interface. Dimers extracted from the interfacial ensemble exhibit nearly symmetric forward and backward CT contributions and, consequently, an almost vanishing net transfer, consistent with recent energy-decomposition analyses.^31^ On the other hand, larger molecular clusters containing up to three decanes and 12 waters show small net CT from decane to water. In striking contrast, the extended interface displays a clear electronic asymmetry, yielding a systematic net CT from water to the hydrocarbon phase. The reduced 2 × 2 electronic *T*-matrix reveals that this net interphase CT represents a small but systematic component of the total response, with substantially larger intraphase electron reorganizations, particularly within the hydrocarbon phase, demonstrating the dominant role of collective many-body polarization. Our atom-resolved analysis relates the CT imbalance to the distinct geometrical distributions of forward C–H···O and backward O–H···C contacts, and specifically to the tendency of interfacial water molecules to accept weak improper hydrogen bonds from hydrocarbon C–H groups. This collective intra- and interphase electronic response is accompanied by subtle contractions of interfacial O–H and C–H covalent bonds, consistent with the characteristic vibrational signatures expected for weak improper hydrogen bonds at oil–water interfaces.

The calculated net CT corresponds to an average surface charge density of σ ∼ 0.003 *e* ^–^ nm^–2^, or approximately 0.5 mC m^–2^, on the hydrocarbon side. This value is larger than previous estimates obtained from models in which CT was restricted to water–water interactions^20^ or from cluster-based ALMO/EDA analyses,^31^ but is smaller than the value reported by Poli and Hassanali using BLYP-based calculations.^28^ This discrepancy originates from the tendency of GGA functionals like BLYP to overdelocalize charge^64^ (see SI for details). Within the assumptions required to relate the measured ζ-potential to a surface charge density, our estimate is also on the same order of magnitude of the experimental value of approximately 0.008 *e* ^–^ nm^–2^ reported for salt-free oil droplets at neutral pH.^20^

More broadly, the proposed density-partitioning and optimal-transport VIPER-CT protocol provides a practical and transferable route for characterizing directional CT, charge redistribution, and self-polarization in complex molecular environments such as extended interfacial systems. Its application to oil–water interfaces shows that the negative charge of oil–water interfaces is an emergent collective property of extended systems, arising from many-body polarization and the structural asymmetry between interfacial C–H···O and O–H···C contacts rather than from isolated finite molecular clusters interactions alone. By reconciling the nearly canceling and opposite in sign CT contributions found in molecular clusters with the systematic water-to-oil electronic imbalance emerging at the extended interface, this work provides a unified framework for interpreting charge separation, electrostatics, and vibrational signatures at complex aqueous-hydrophobic interfaces.

## Supplementary Material



## Data Availability

The VIPER-CT code is available at https://github.com/Xgabri11X/VIPER-CT. Data for this article, including the DPMD configurations used for the whole interface, decane-only, and water-only systems that are needed to execute the electronic-structure self-consistent-field calculations, are available on Zenodo under 10.5281/zenodo.20159191.

## Notes

Electron density partitioning tests, a quantum benchmark of charge transfer in the water dimer, *T*-matrices for microsolvated decane-water clusters, density-derived atomic point charges (DDAPC) of the full oil–water slab, dipole analysis from Maximally Localized Wannier Functions (MLWFs) of the extended decane-water interface, contact-resolved C–H bond contraction at the interface, a DFT benchmark of the extended decane-water interface, and interaction energies for water-decane dimers and at the extended interface (PDF)
