# Supplementary material for: Mapping Collective Electron-Density Rearrangements at Oil–Water Interfaces Using Optimal Transport
Source: J Chem Theory Comput. 2026 Oct 21;22(21):11583–94. doi: 10.1021/acs.jctc.6c01556 (PMC13757754; doi:10.1021/acs.jctc.6c01556)
Supplement: Supplementary file 1 [file ct6c01556_si_001.pdf]

Supporting Information
Mapping Collective Electron-Density Rearrangements at Oil-Water Interfaces Using Optimal Transport

Gabriele Amante,[†,‡] Klaudia Mráziková,[†,¶] Gabriele Centi,[‡] Sylvie Roke,[§] Ali Hassanali,[*,‖] and Giuseppe Cassone[*,†]

†*Institute for Chemical-Physical Processes, National Research Council (CNR-IPCF), V.le F. Stagno d'Alcontres 37, 98158 Messina, Italy*

‡*Department of Chemical, Biological, Pharmaceutical, and Environmental Sciences, University of Messina, Viale F. Stagno d'Alcontres 31, 98166 Messina, Italy*

¶*J. Heyrovský Institute of Physical Chemistry, Academy of Sciences of the Czech Republic, Dolejškova 3, CZ-18223 Prague 8, Czech Republic*

§*Laboratory for Fundamental BioPhotonics (LBP), Institute of Bioengineering, École Polytechnique Fédérale de Lausanne (EPFL), 1015 Lausanne, Switzerland*

‖*International Centre for Theoretical Physics (ICTP), Strada Costiera 11, 34151 Trieste, Italy*

E-mail: ahassana@ictp.it; giuseppe.cassone@cnr.it

Table S1: Per-atom electron populations of the gas-phase water dimer optimized at the MP2/aug-cc-pVQZ theory level and electron density determined at the CCSD/aug-cc-pVTZ level. Integrated electron density assigned to each atomic basin, $N_e(A) = \int_{\Omega_A} \rho(\mathbf{r})\, d\mathbf{r}$ (in electrons), computed on the dimer using (i) a pure Voronoi tessellation and (ii) a vdW-weighted Voronoi (power-diagram) tessellation. Atoms are grouped into the hydrogen-bond donor (d subscript) and acceptor (a subscript) monomers. H_d^{HB} denotes the hydrogen atom directly involved in hydrogen bonding.

Monomer	Atom label	N_e (Voronoi)	N_e (vdW–Voronoi)
Mon1	O_d	7.3720	8.1613
Mon1	$\mathrm{H}_d^{\mathrm{HB}}$	1.4324	0.9618
Mon1	H_d	1.3628	0.9764
Mon2	O_a	7.2065	8.0301
Mon2	H_a	1.3333	0.9519
Mon2	H_a	1.3330	0.9610

Electron Density Partitioning

To benchmark the capabilities of the proposed density-difference VIPER-CT (Voronoi-Informed Partitioning of Electron Redistribution for Charge Transfer) partitioning scheme, we first consider molecular dimers, a class of systems for which extensive reference literature is available. As a prototypical case, we study the gas-phase water dimer. We optimize its geometry at the MP2/aug-cc-pVQZ level to the well-known ground-state non-planar configuration[1] and then compute the electron density at the CCSD/aug-cc-pVTZ for three fragments: the full dimer, the hydrogen-bond donor monomer, and the hydrogen-bond acceptor monomer. We subsequently evaluate the integral in eq. 3 of the main text on the dimer density and assign the resulting electron population to atom-centered basins using two alternative tessellations: (i) a *pure* Voronoi construction, where each grid point is assigned to the nearest nucleus, and (ii) a *vdW-weighted* Voronoi (power-diagram) construction, where distances are scaled by the atomic van der Waals radii. The corresponding per-atom electron populations, $N_e(A) = \int_{\Omega_A} \rho(\mathbf{r})\, d\mathbf{r}$, are reported in Table S1. Table S1 illustrates a systematic bias of the pure Voronoi partitioning: it assigns too few electrons to oxygen and too many to hydrogen. This behavior is expected because a nearest-nucleus Voronoi tessellation implicitly treats all atoms as having comparable spatial extent; as a result, it under-allocates volume to larger atoms (oxygen) and over-allocates it to smaller ones (hydrogen), distorting the in-

tegrated electron populations. As visible from the last column of Table S1, introducing the vdW weighting corrects this geometric mismatch by expanding the effective basin of oxygen relative to hydrogen, thereby reallocating electron density to the chemically appropriate centers. For this reason, in the VIPER-CT framework we adopt the vdW-weighted Voronoi (power-diagram) construction as our default partitioning scheme.

Charge Transfer in the Water Dimer

We now apply the vdW-weighted Voronoi (power-diagram) VIPER-CT partitioning to the density difference

$$\Delta\rho(\mathbf{r}) = \rho_{\mathrm{dimer}}(\mathbf{r}) - \rho_{\mathrm{mon1}}(\mathbf{r}) - \rho_{\mathrm{mon2}}(\mathbf{r}), \tag{1}$$

and quantify how much electron density is displaced upon dimer formation. For each atomic basin Ω_A we compute the electron-gain/loss contributions

$$\Delta N_e^+(A) = \int_{\Omega_A} \max\big(\Delta\rho(\mathbf{r}), 0\big)\, d\mathbf{r}, \tag{2}$$

$$\Delta N_e^-(A) = \int_{\Omega_A} \min\big(\Delta\rho(\mathbf{r}), 0\big)\, d\mathbf{r}, \tag{3}$$

$$\Delta N_e(A) = \Delta N_e^+(A) + \Delta N_e^-(A) = \int_{\Omega_A} \Delta\rho(\mathbf{r})\, d\mathbf{r}, \tag{4}$$

where $\Delta N_e(A)$ (in electrons) is the net electron accumulation (> 0) or depletion (< 0) on atom A induced by the interaction. The monomer-resolved CT is then obtained by summing $\Delta N_e(A)$ over the atoms belonging to the hydrogen-bond donor or hydrogen-bond acceptor water, respectively. From Table S2, the hydrogen-bond donor monomer – whose atoms are marked by the subscript d – gains $\approx 0.01330\ e^-$ while the hydrogen-bond acceptor loses $\approx 0.01327\ e^-$, with the residual non-zero total ($\sim 3 \times 10^{-5}\ e^-$) reflecting a small numerical integration/partitioning noise.

To enable a direct comparison with the benchmark analysis by Belpassi, Ronca and co-workers,[2] we adopt the same computational protocol for the reference electronic densities:

Table S2: vdW-weighted Voronoi analysis of $\Delta\rho(\mathbf{r})$ determined for the water dimer optimized at the MP2/aug-cc-pVQZ theory level and electron density determined at the CCSD/aug-cc-pVTZ level. Per-atom integrals of the positive and negative parts of the density difference, ΔN_e^+ and ΔN_e^-, and net electron accumulation $\Delta N_e = \int_{\Omega_A} \Delta\rho(\mathbf{r})\, d\mathbf{r}$ (in e), obtained with the vdW-weighted Voronoi (power-diagram) partitioning. Atoms are grouped into H-bond donor and acceptor monomers; H_d^{HB} denotes the donor hydrogen pointing toward the H-bond acceptor oxygen atom. Monomers' CT is computed as the sum of ΔN_e over atoms belonging to each monomer.

Monomer	Atom label	ΔN_e^+ (e)	ΔN_e^- (e)	ΔN_e (e)
Mon1	O_d	0.04479	-0.01132	0.03347
Mon1	H_d^{HB}	0.00113	-0.03003	-0.02890
Mon1	H_d	0.00919	-0.00047	0.00873
	Mon1 sum (CT)	0.05512	-0.04182	0.01330
Mon2	O_a	0.02343	-0.01359	0.00984
Mon2	H_a	0.00014	-0.01173	-0.01159
Mon2	H_a	0.00018	-0.01171	-0.01153
	Mon2 sum (CT)	0.02375	-0.03702	-0.01327
	Total (dimer)	0.07886	-0.07884	0.00003

the nuclear coordinates are optimized at the MP2/aug-cc-pVQZ level, and the electron densities entering $\Delta\rho(\mathbf{r})$ are evaluated at the CCSD/aug-cc-pVTZ level, employing the full dimer basis also for the monomers (counterpoise-like treatment to control BSSE). Under these matched conditions, the net CT we evaluate $\approx 0.0133\ e^-$ is in close agreement with the value of $0.0146\ e^-$ reported in Ref.[2]

Inspecting Table S2 reveals that the electronic rearrangement induced by dimer formation is not evenly distributed between the two monomers. Indeed, summing the magnitudes of the positive and negative contributions, $q_{\text{tot}}(\text{mon}) \equiv \sum_{A\in\text{mon}} \left(\Delta N_e^+(A) + |\Delta N_e^-(A)|\right)$, shows that the hydrogen-bond donor undergoes a larger overall density reorganization than the hydrogen-bond acceptor (i.e., $q_{\text{tot}}^{\text{mon1}} = 0.09694\ e^-$ vs. $q_{\text{tot}}^{\text{mon2}} = 0.06077\ e^-$). This observation already suggests that, besides the net inter-monomer CT, a substantial fraction of the response is associated with intra-monomer self-polarization.

To disentangle these two contributions in a rigorous and transferable way, we solve the optimal-transport problem defining the T-matrix (see the main text), which maps the electron-loss distribution ($-\Delta\rho$) onto the electron-gain distribution ($+\Delta\rho$) while minimizing the prescribed distance cost. Coarse-graining the resulting atom-resolved matrix T_{ij} over the

Table S3: Electronic coarse-grained 2×2 T-matrix of the gas-phase water dimer optimized at the MP2/aug-cc-pVQZ level and whose electron densities are evaluated at the CCSD/aug-cc-pVTZ theory. Diagonal elements correspond to self-polarization terms while the off-diagonal elements correspond to the inter-monomer CT. Entries are in electrons (e^-).

	Electron donor	
Electron acceptor	Monomer1	Monomer2
Monomer1	0.04182	0.01328
Monomer2	0.00000	0.02374

two monomers yields the 2×2 transport matrix reported in Table S3. In this representation, diagonal elements quantify self-polarization within each monomer (electron depletion and accumulation occurring within the same fragment), whereas off-diagonal elements quantify genuine inter-monomer CT.

Consistently with the net ΔN_e balance in Table S2, the off-diagonal term $T_{mon2 \to mon1} = 0.01328\ e^-$ captures the CT from the hydrogen-bond acceptor (electron source) to the hydrogen-bond donor (electron receiver) producing a net accumulation of extra electronic charge on the hydrogen-bond donor water molecule. The diagonal entries show that self-polarization dominates the response: $T_{mon1 \leftrightarrow mon1} = 0.04182\ e^-$ and $T_{mon2 \leftrightarrow mon2} = 0.02374\ e^-$, confirming that the hydrogen-bond donor performs most of the "electronic work" in accommodating the interaction, with an intra-fragment rearrangement that is nearly twice as large as that of the hydrogen-bond acceptor. Taken together, Tables S2 and S3 demonstrate that the present VIPER-CT framework not only recovers a realistic inter-monomer CT magnitude, but also quantitatively separates it cleanly from the larger, fragment-local polarization component.

Our goal is not only to reproduce a reliable CT magnitude on a small benchmark (the gas-phase water dimer), but to obtain a well-converged self-consistent-field (SCF) electron density for an extended heterogeneous system (i.e., a decane-water slab), from which $\Delta\rho$ and the coarse-grained transport matrix can be computed consistently. While CCSD(T)[3–6] calculations provide excellent references on small clusters, these calculations are computationally prohibitive for the periodic systems one intends to investigate for measuring collective,

Table S4: Gas-phase water dimer: inter-monomer charge transfer summary. Values report the off-diagonal coarse-grained transport-matrix element $T_{mon2 \rightarrow mon1}$ (CT magnitude), in electrons (e^-). Columns indicate the geometry-optimization level; rows indicate the level used to compute the electron density / CT.

	Geometry optimization level		
Electron density / CT level	ωB97X	M06-2X	CCSD(T)
BLYP+D3	0.02108	0.02158	0.02158
r2SCAN	0.01952	0.02007	0.02007
PBE0	0.01879	0.01918	0.01918
CCSD(T)	0.01245	0.01219	0.01219
ωB97X	0.01795	0.01807	0.01807

many-body electronic effects. Likewise, hybrid and range-separated hybrid functionals become significantly more expensive for large clusters (or periodic models), making systematic density calculations across many configurations impractical.

In this context, r2SCAN[7] offers an excellent compromise between accuracy, numerical robustness, and scalability. As a modern meta-GGA (a regularized variant of SCAN[8]), r2SCAN typically provides stable SCF convergence and reduced sensitivity to numerical settings (e.g., integration grids) compared to SCAN, while retaining a much improved description over standard GGAs. This balance is particularly relevant for water-hydrocarbon complexes, where hydrogen bonding and dispersion-driven interactions must be described simultaneously without incurring the cost of exact exchange.

The water-dimer benchmark we report in Table S4 indicates that the inter-monomer CT, quantified by the off-diagonal coarse-grained element $T_{mon2 \rightarrow mon1}$, is only weakly affected by the optimization level when computed with r2SCAN. Across the tested geometries, r2SCAN yields $T_{mon2 \rightarrow mon1}$ values in a narrow range (approximately 0.0195–0.0201 e^-), pointing to a robust and internally consistent density response. This limited sensitivity is important for extended systems, where an ensemble of molecular configurations is unavoidable and the analysis requires a method that does not introduce spurious fluctuations in $\Delta\rho$ or derived CT metrics. Although the CCSD(T) predicts more conservative estimates of the CT in the water dimer, values predicted by r2SCAN are in fairly good agreement with those evaluated

a) **2 water molecules**

	Electron donor	
Electron acceptor	Decane	Water
Decane	0.05391	0.00063
Water	0.00304	0.00948

b) **4 water molecules**

	Electron donor	
Electron acceptor	Decane	Water
Decane	0.07543	0.00120
Water	0.00310	0.01604

c) **6 water molecules**

	Electron donor	
Electron acceptor	Decane	Water
Decane	0.08594	0.00129
Water	0.00319	0.02045

d) **8 water molecules**

	Electron donor	
Electron acceptor	Decane	Water
Decane	0.08939	0.00121
Water	0.00302	0.02359

Figure S1: Average 2×2 electronic T-matrices for one decane molecule microsolvated by 2, 4, 6, and 8 water molecules. Rows denote electron acceptors and columns electron donors. The off-diagonal imbalance remains directed from decane toward water for all displayed hydration numbers.

by the ωB97X[9] and PBE0[10] exchange and correlation functionals, as displayed in Table S4.

Microsolvated Decane-Water Clusters: Hydration and Finite-Size Dependence

To test whether the direction of the CT imbalance evolves continuously from dimers toward the extended interface, we constructed microsolvated clusters extracted from the same DPMD trajectory. The cluster series contains one, two, or three decane molecules together with an increasing number of surrounding waters. For the one-decane series, 100 configurations were analyzed for each hydration number $N_{H_2O} = 2, 4, 6,$ and 8; for the two- and three-decane series, 50 configurations were analyzed for each $N_{H_2O} = 2, 4, 6, 8, 10,$ and 12. For every cluster, the full and frozen isolated-fragment densities were recomputed and analyzed with the same vdW-weighted Voronoi and optimal-transport protocol used throughout the manuscript.

a) 2 water molecules

	Electron donor	
Electron acceptor	Decanes	Water
Decanes	0.07632	0.00104
Water	0.00462	0.01183

b) 4 water molecules

	Electron donor	
Electron acceptor	Decanes	Water
Decanes	0.10558	0.00140
Water	0.00533	0.02012

c) 6 water molecules

	Electron donor	
Electron acceptor	Decanes	Water
Decanes	0.12169	0.00179
Water	0.00511	0.02617

d) 8 water molecules

	Electron donor	
Electron acceptor	Decanes	Water
Decanes	0.13290	0.00189
Water	0.00535	0.03131

e) 10 water molecules

	Electron donor	
Electron acceptor	Decanes	Water
Decanes	0.13839	0.00156
Water	0.00543	0.03494

f) 12 water molecules

	Electron donor	
Electron acceptor	Decanes	Water
Decanes	0.14534	0.00168
Water	0.00564	0.03805

Figure S2: Average 2×2 electronic T-matrices for two decane molecules microsolvated by 2–12 water molecules. Increasing hydration enhances the diagonal self-polarization terms but does not induce a water-to-decane net CT.

a) 2 water molecules

	Electron donor	
Electron acceptor	Decanes	Water
Decanes	0.08612	0.00106
Water	0.00470	0.01216

b) 4 water molecules

	Electron donor	
Electron acceptor	Decanes	Water
Decanes	0.12202	0.00143
Water	0.00544	0.02085

c) 6 water molecules

	Electron donor	
Electron acceptor	Decanes	Water
Decanes	0.14283	0.00198
Water	0.00522	0.02716

d) 8 water molecules

	Electron donor	
Electron acceptor	Decanes	Water
Decanes	0.15712	0.00215
Water	0.00557	0.03259

e) 10 water molecules

	Electron donor	
Electron acceptor	Decanes	Water
Decanes	0.16657	0.00179
Water	0.00561	0.03681

f) 12 water molecules

	Electron donor	
Electron acceptor	Decanes	Water
Decanes	0.17710	0.00192
Water	0.00619	0.04045

Figure S3: Average 2×2 electronic T-matrices for three decane molecules microsolvated by 2–12 water molecules. As for the one- and two-decane clusters, the small off-diagonal imbalance remains directed toward water.

The one-decane clusters exhibit a mean CT toward water between 0.00198 and 0.00254 e^- across the investigated hydration numbers. The corresponding ranges are 0.00355–0.00414 e^- for two decanes and 0.00349–0.00447 e^- for three decanes. Averaging over hydration yields 0.00219, 0.00387, and 0.00394 e^- for one, two, and three decanes, respectively. Thus, although the total density rearrangement and self-polarization grow as the cluster becomes larger, no crossover toward the water→decane direction is observed. This result directly

addresses the finite-size question: the local asymmetry of the interfacial C–H$\cdots$O/O–H$\cdots$C contacts is already present in the extracted clusters, yet it does not reproduce the sign of the extended-interface CT. Finite microsolvation is therefore insufficient, within the cluster sizes tested here, to generate the slab asymmetry.

Inspection of the matrices in Figs. S1–S3 additionally shows that the dominant size dependence occurs in the diagonal terms. The decane self-polarization grows steadily as waters and additional hydrocarbons are included, and the water self-polarization increases with hydration as well. In contrast, the two off-diagonal terms remain at the 10^{-3}–10^{-2} e^- scale and preserve the same small net imbalance toward water. The finite clusters therefore become electronically more polarizable without approaching the direction of the slab CT.

Independent Periodic Charge and Water-Dipole Benchmarks

Density-Derived Atomic Point Charges

As an independent population analysis of the periodic system, we evaluated the density-derived atomic point charges (DDAPC)[11] available from the converged CP2K electronic densities. Unlike the deformation-density T-matrix of our VIPER-CT approach, which is defined relative to the frozen isolated subsystems, DDAPC partitions the total ground-state density into atom-centered point charges. We therefore use it as a sign and phase-charge cross-check rather than expecting numerical equality with the interaction-induced CT.

For each configuration, the atomic DDAPC values were summed over all atoms belonging to the water and decane phases,

$$Q_{\mathrm{water}}^{\mathrm{DDAPC}} = \sum_{i\in\mathrm{water}} q_i^{\mathrm{DDAPC}}, \qquad Q_{\mathrm{decane}}^{\mathrm{DDAPC}} = \sum_{i\in\mathrm{decane}} q_i^{\mathrm{DDAPC}}. \tag{5}$$

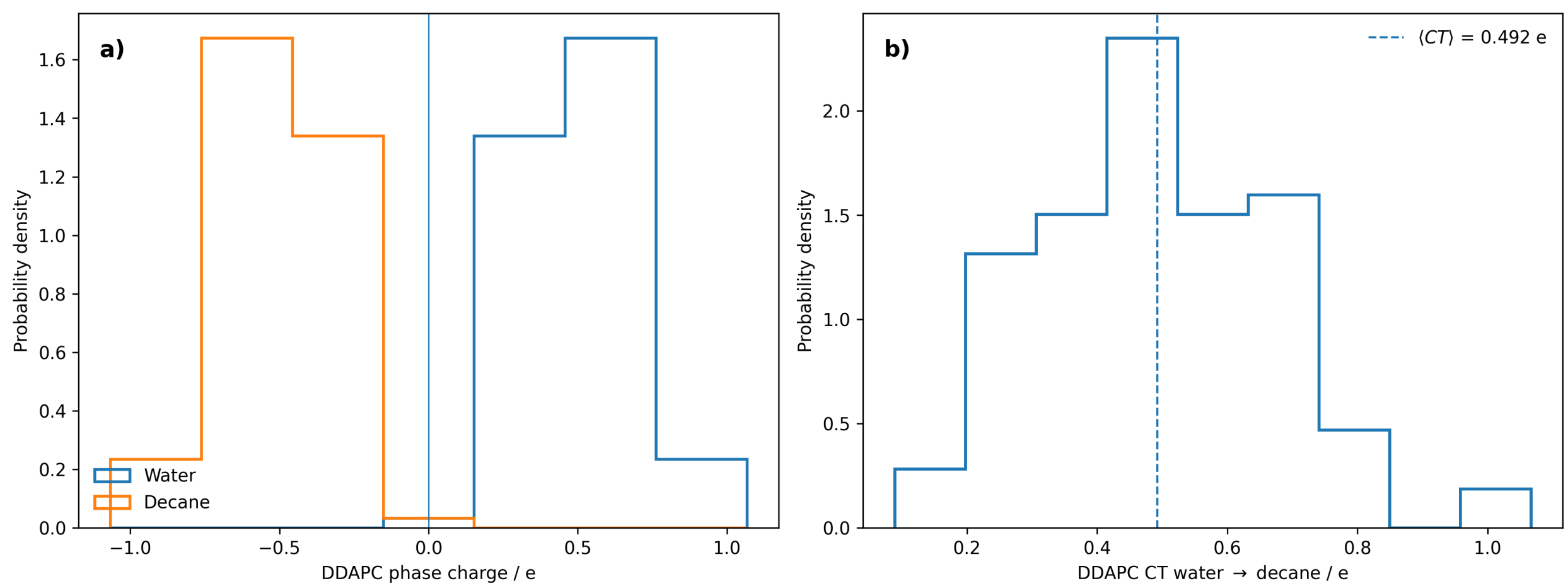

Figure S4: (a) Distributions of the total DDAPC charge assigned to the water (blue line) and decane (orange line) phases. (b) Magnification of the distribution of Q_{water}, plotted using the relevant range of charges and a thinner binning.

Across 100 converged configurations, we obtain

$$\langle Q_{\mathrm{water}}^{\mathrm{DDAPC}} \rangle = +0.4922 \pm 0.1916\ e^{-}, \qquad \langle Q_{\mathrm{decane}}^{\mathrm{DDAPC}} \rangle = -0.4922 \pm 0.1916\ e^{-}, \tag{6}$$

where the uncertainties are snapshot-to-snapshot standard deviations. The mean total-charge residual is $-3.98 \times 10^{-7}\ e^{-}$ with a standard deviation of $9.20 \times 10^{-6}\ e^{-}$. Thus, a fully independent periodic partial-charge partition assigns the hydrocarbon phase a negative average charge, in agreement with the sign obtained from the $\Delta\rho$ analysis. The larger absolute DDAPC magnitude is not interpreted as a direct CT value because the two definitions answer different questions.

The distributions in Fig. S4 show that this assignment is not produced by a small subset of anomalous configurations: the phase charges fluctuate substantially but are centered on opposite signs, and the total water+decane charge remains essentially zero. The DDAPC analysis therefore provides a robust periodic sign-level validation while also illustrating why absolute partial-charge magnitudes should not be compared one-to-one with deformation-density CT values.

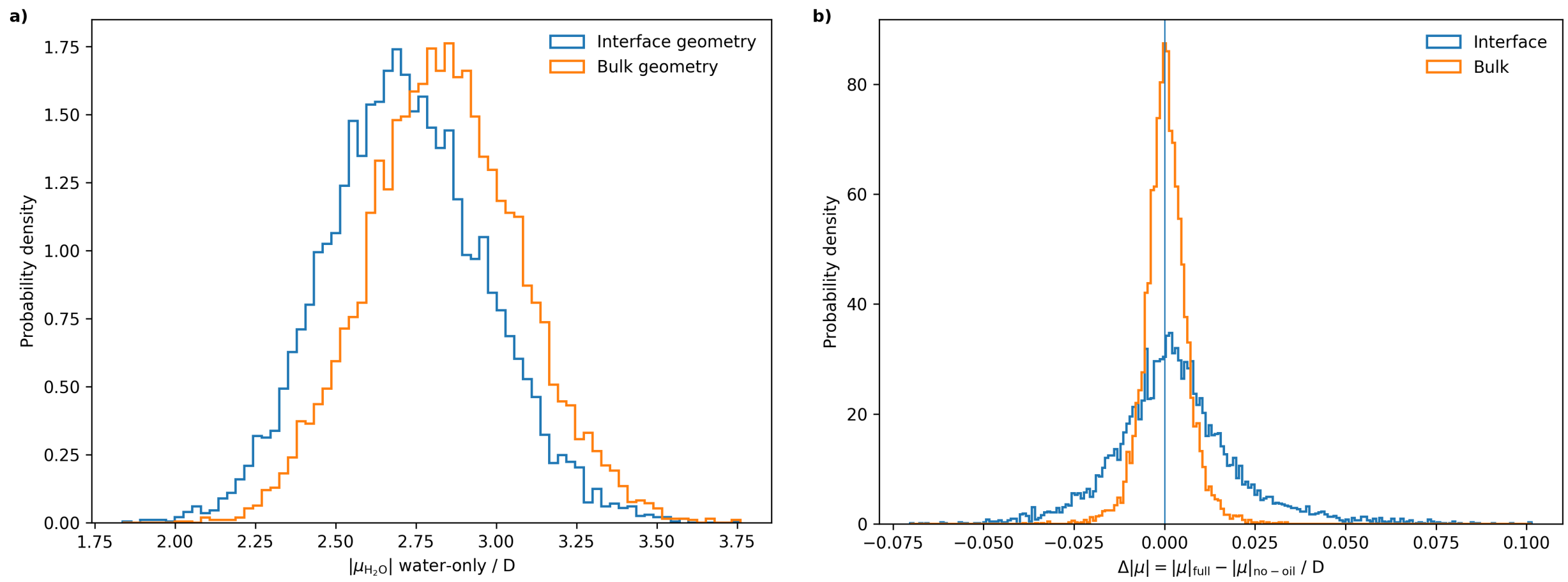

Figure S5: (a) Absolute water-only maximally localized Wannier centers dipole distributions for molecules retaining interfacial (blue line) and bulk-like (orange line) nuclear geometries in absence of the oil phase in the *ab initio* re-sampling. (b) Distribution of the paired dipole change $\Delta|\mu| = |\mu|_{\mathrm{full}} - |\mu|_{\mathrm{water-only}}$ for interfacial (blue line) and bulk-like (orange line) waters.

Individual Water Dipoles from Maximally Localized Wannier Functions

For each water molecule, four doubly occupied maximally localized Wannier centers[12] were associated with the corresponding oxygen. Using the valence ionic charges of the GTH pseudopotentials,[13] the molecular dipole was evaluated as

$$\boldsymbol{\mu}_{\mathrm{H_2O}} = 6\mathbf{r}_{\mathrm{O}} + \mathbf{r}_{\mathrm{H}_1} + \mathbf{r}_{\mathrm{H}_2} - 2\sum_{n=1}^{4} \mathbf{r}_{W_n}, \tag{7}$$

with periodic coordinates unwrapped around the oxygen and the result converted to Debye. The same water geometries were then analyzed after removing the decane phase, providing a paired water-only electronic reference. Interfacial and bulk-like waters were classified from the instantaneous Willard-Chandler surface using the same $|d_{\mathrm{WC}}| \leq 3.0$ Å criterion adopted in the main analysis.

These two comparisons separate structural and electronic contributions. First, the water-only calculation retains the nuclear geometry sampled at the oil-water interface; the difference between the interfacial-geometry and bulk-geometry distributions displayed in Fig.

Table S5: C–H bond-length statistics for interfacial C–H groups classified as participating or not participating in C–H$\cdots$O hydrogen-bond-like contacts.

Population	N	Fraction (%)	$\langle r_{\mathrm{CH}} \rangle$ (Å)	$\sigma(r_{\mathrm{CH}})$ (Å)
HB-like	21,539,991	16.487	1.0812	0.0701
non-HB	109,107,272	83.513	1.0896	0.0990

S5-a therefore reflects a geometrical/coordination contribution. Second, comparing each water with and without decane at fixed coordinates isolates the additional electronic response induced by the oil phase. As shown in Fig. S5-b, the substantially broader $\Delta|\mu|$ distribution at the interface indicates that this direct electronic contribution is heterogeneous and concentrated near the boundary.

More specifically, the absolute water-only distribution for interfacial geometries is shifted toward lower dipole magnitudes relative to bulk-like geometries, indicating that the altered interfacial nuclear structure by itself tends to reduce the molecular polarization. Restoring decane does not simply shift all interfacial dipoles in one direction: the paired $\Delta|\mu|$ distribution develops both positive and negative tails. Hence the oil-induced contribution is best described as a broadening and heterogeneous redistribution of local water polarization, consistent with a fluctuating many-body interfacial environment rather than a uniform pairwise perturbation.

Contact-Resolved C–H Bond Contraction

To determine whether the C–H contraction discussed in the main text is specifically associated with the forward C–H$\cdots$O contact motif, we separated interfacial C–H bonds into hydrogen-bond-like and non-hydrogen-bonded populations using the same geometrical classification employed for the interfacial motif analysis. The resulting statistics are summarized in Table S5.

The hydrogen-bond-like population has an average C–H bond approximately 0.0084 Å shorter than the non-hydrogen-bonded interfacial population. Thus, the contraction ob-

served in the distance-to-interface average is not merely a uniform shift affecting all interfacial hydrocarbon groups: it is specifically enhanced among the C–H groups participating in the contact motif implicated in the charge-rearrangement analysis. This strengthens the structural connection to the experimentally observed blue-shifted C–H feature.

Benchmark on the Extended Decane-Water Interface

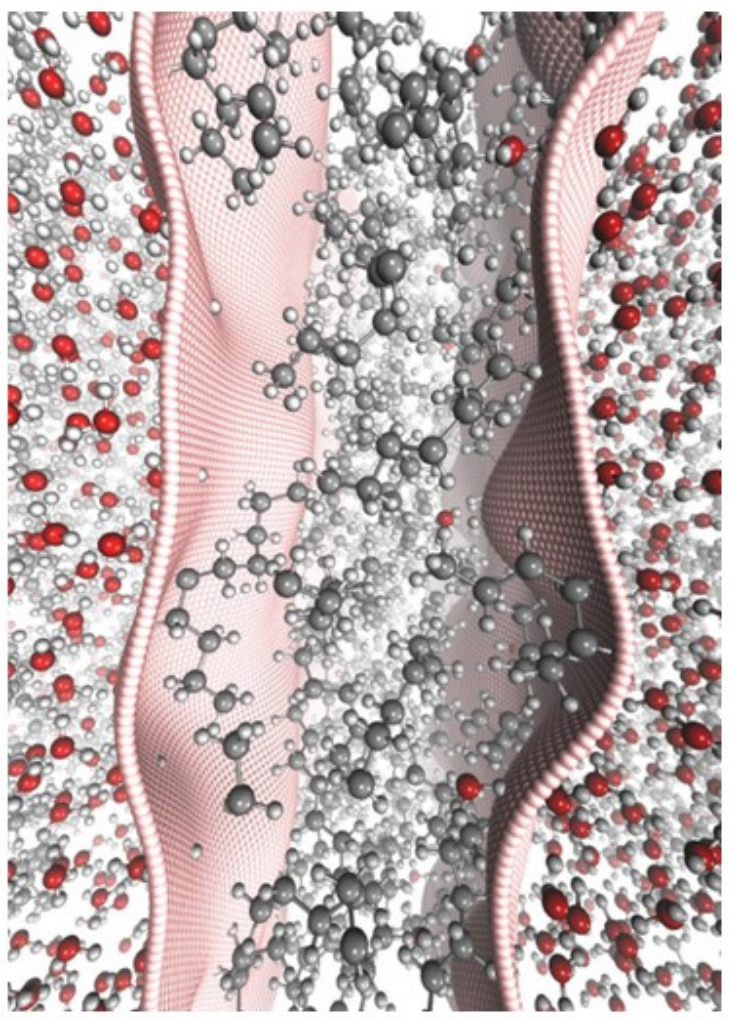

BLYP+D3

	Electron donor	
Electron acceptor	Decane	Water
Decane	0.64714	0.10396
Water	0.00114	0.08303

SCAN

	Electron donor	
Electron acceptor	Decane	Water
Decane	0.65620	0.03260
Water	0.00741	0.10023

r2SCAN

	Electron donor	
Electron acceptor	Decane	Water
Decane	0.65673	0.03152
Water	0.00776	0.10031

Method	Net CT (e^-)
BLYP+D3	0.10282
SCAN	0.02519
r2SCAN	0.02376

Figure S6: Benchmark of charge transfer (CT) at the extended decane-water interface across different density functional approximations. Representative snapshot of the decane-water slab is displayed on the left; the pink surface highlights the instantaneous interfacial region used to define the two phases. On the right, optimal-transport T-matrices obtained from the interaction-induced density rearrangement $\Delta\rho(\mathbf{r})$ using the vdW-weighted Voronoi partitioning, shown for BLYP+D3, SCAN, and r2SCAN. Columns label the electron donor phase and rows the electron acceptor phase; diagonal elements quantify intra-phase charge rearrangement (self-polarization), while off-diagonal elements quantify cross-phase CT. Numbers are in units of electrons. The last non-colored Table shows the net CT, defined as forward minus backward transfer (water→decane minus decane→water), for each method.

Figure S6 reports the fragment-level (2×2) coarse-grained transport T-matrix obtained by summing the atom-resolved optimal-transport matrix over the decane and water fragments. Diagonal entries quantify intra-fragment charge rearrangement (self-polarization), whereas off-diagonal entries quantify inter-fragment charge transfer (CT). Within this convention, the net CT from water to decane is given by $\mathrm{CT}_{\mathrm{net}} = T_{\mathrm{Water}\to\mathrm{Decane}} - T_{\mathrm{Decane}\to\mathrm{Water}}$, and is summarized in the right-hand non-colored Table.

All three matrices are dominated by the diagonal terms, indicating that the leading response at the interface is polarization within each fragment rather than genuine elec-

tron transfer across fragments. However, the magnitude of the off-diagonal contribution is strongly functional-dependent. BLYP+D3[14–17] predicts a relatively large $T_{\text{Water}\rightarrow\text{Decane}}$ (and thus a large net CT, $\sim 0.103\ e^-$), whereas both SCAN and r2SCAN yield substantially smaller values (net CT $\sim$ 0.024–0.025 e^-), while remaining mutually consistent. To provide a comparison with the surface charge density reported in the main text, the BLYP+D3 estimate of $\sigma = \frac{CT_{net}}{L_x \times L_y} = 0.02670\ e^-\,\text{nm}^{-2} \sim 4.3\ \text{mC}\,\text{m}^{-2}$, where $L_{x,y}$ are the simulation box edges, is a factor of ~ 4 larger than the SCAN/r2SCAN predictions which locate at $\sim 1.0\ \text{mC}\,\text{m}^{-2}$. The fact that SCAN and r2SCAN agree closely suggests that the smaller CT is a robust feature of the meta-GGAs description of the interface, whereas the larger BLYP+D3 value is consistent with the well-known tendency of semilocal GGAs to over-delocalize charge (self-interaction/delocalization error),[18] which enhances CT in weakly bound heterogeneous complexes.

We therefore selected r2SCAN as the working level for the extended decane-water slab. r2SCAN is a regularized meta-GGA, which in practice delivers more stable and reproducible SCF convergence (reduced sensitivity to numerical settings) than SCAN on large, heterogeneous systems, an essential requirement when computing electron densities for many snapshots of an extended interface. Finally, as a semilocal functional, it preserves the computational efficiency needed at this scale, enabling systematic, density-based analyses in realistic slab models involving a thousand of atoms and a thousand of configurations, that are not tractable with wavefunction methods and are considerably more demanding with hybrid/range-separated functionals.

Interaction Energies of Water-Decane Dimers and at the Extended Interface

Figure S7 reports the distribution of the interaction energies computed at the r2SCAN level of theory for both the water-decane dimer dataset and the extended interface model. As for the

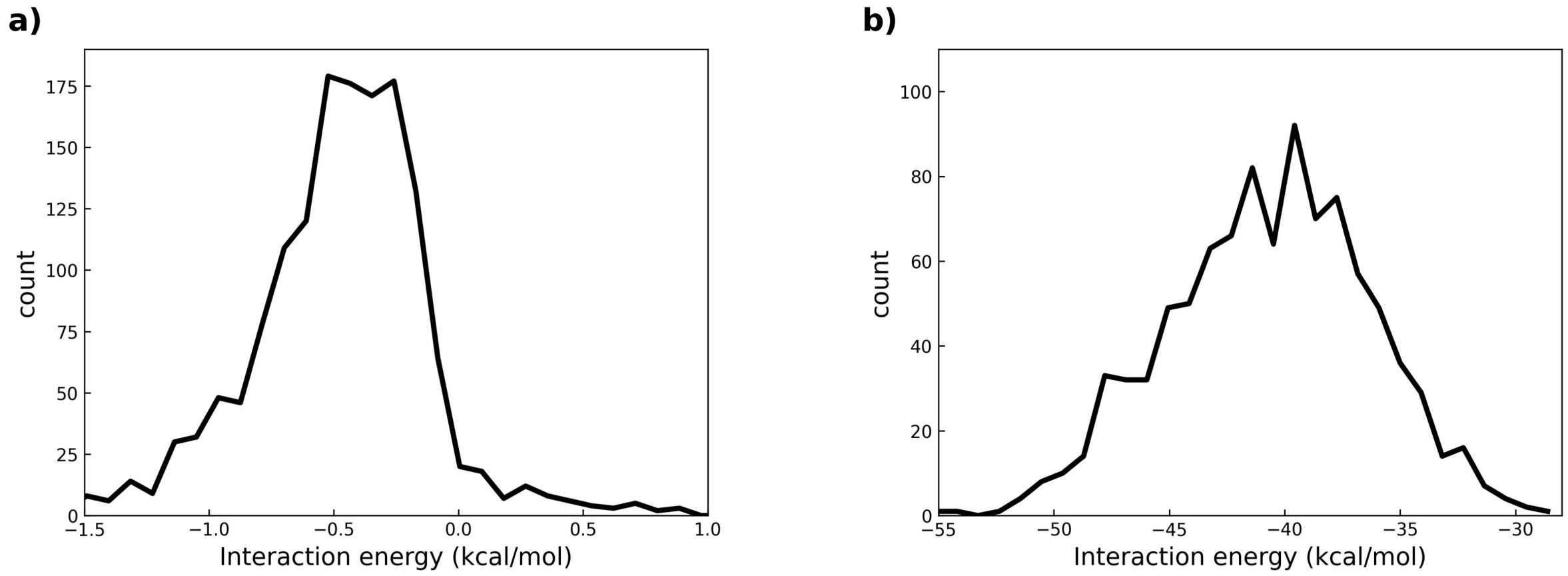

Figure S7: Distribution of the interaction energies computed at the r2SCAN level of theory. (a) 1500 water-decane dimers extracted from the DPMD trajectory; (b) extended water-decane interface of the same DPMD trajectory.

1500 water-decane dimers (Figure S7a), the distribution is centered around ~ -0.5 kcal/mol and spans a relatively broad range from approximately -1.5 to $+1.0$ kcal/mol. Notably, a non-negligible fraction of dimer configurations exhibits positive interaction energies, indicating the (locally) repulsive nature of some dimers' arrangements sampled during the DPMD trajectory. In net contrast, the extended interface model (Figure S7b) displays a distribution entirely shifted to negative values, centered around ~ -40 kcal/mol and ranging from approximately -55 to -28 kcal/mol. The absence of positive interaction energies is another indicator of the cooperative and many-body nature of the physics behind the stabilization of the extended system, where the cumulative effect of multiple intermolecular contacts ensures a net, global, stabilizing interaction across all sampled molecular configurations.

References

(1) Lane, J. R. CCSDTQ Optimized Geometry of Water Dimer. *J. Chem. Theory. Comput.* **2013**, *9*, 316–323.

(2) Ronca, E.; Belpassi, L.; Tarantelli, F. A Quantitative View of Charge Transfer in the Hydrogen Bond: The Water Dimer Case. *ChemPhysChem* **2014**, *15*, 2682–2687.

(3) Čížek, J. *Advances in Chemical Physics*; John Wiley & Sons, Ltd, 1969; pp 35–89.

(4) Purvis, I., George D.; Bartlett, R. J. A full coupled-cluster singles and doubles model: The inclusion of disconnected triples. *The Journal of Chemical Physics* **1982**, *76*, 1910–1918.

(5) Scuseria, G. E.; Janssen, C. L.; Schaefer, I., Henry F. An efficient reformulation of the closed-shell coupled cluster single and double excitation (CCSD) equations. *The Journal of Chemical Physics* **1988**, *89*, 7382–7387.

(6) Scuseria, G. E.; Schaefer, I., Henry F. Is coupled cluster singles and doubles (CCSD) more computationally intensive than quadratic configuration interaction (QCISD)? *The Journal of Chemical Physics* **1989**, *90*, 3700–3703.

(7) Furness, J. W.; Kaplan, A. D.; Ning, J.; Perdew, J. P.; Sun, J. Correction to "Accurate and Numerically Efficient r2SCAN Meta-Generalized Gradient Approximation". *J. Phys. Chem. Lett.* **2020**, *11*, 9248.

(8) Sun, J.; Ruzsinszky, A.; Perdew, J. P. Strongly Constrained and Appropriately Normed Semilocal Density Functional. *Physical Review Letters* **2015**, *115*, 036402.

(9) Chai, J.-D.; Head-Gordon, M. Long-range corrected hybrid density functionals with damped atom–atom dispersion corrections. *Journal of Chemical Physics* **2008**, *128*, 084106.

(10) Adamo, C.; Barone, V. Toward reliable density functional methods without adjustable parameters: The PBE0 model. *Journal of Chemical Physics* **1999**, *110*, 6158–6170.

(11) Blöchl, P. E. Electrostatic decoupling of periodic images of plane-wave-expanded densities and derived atomic point charges. *The Journal of Chemical Physics* **1995**, *103*, 7422–7428.

(12) Marzari, N.; Vanderbilt, D. Maximally localized generalized Wannier functions for composite energy bands. *Phys. Rev. B* **1997**, *56*, 12847–12865.

(13) Krack, M. Pseudopotentials for H to Kr optimized for gradient-corrected exchange-correlation functionals. *Theoretical Chemistry Accounts* **2005**, *114*, 145–152.

(14) Becke, A. D. Density-functional exchange-energy approximation with correct asymptotic behavior. *Phys. Rev. A* **1988**, *38*, 3098–3100.

(15) Lee, C.; Yang, W.; Parr, R. G. Development of the Colle-Salvetti correlation-energy formula into a functional of the electron density. *Phys. Rev. B* **1988**, *37*, 785–789.

(16) Grimme, S.; Antony, J.; Ehrlich, S.; Krieg, H. A consistent and accurate ab initio parametrization of density functional dispersion correction (DFT-D) for the 94 elements H-Pu. *J. Chem. Phys.* **2010**, *132*, 154104.

(17) Grimme, S.; Ehrlich, S.; Goerigk, L. Effect of the damping function in dispersion corrected density functional theory. *J. Comput. Chem.* **2011**, *32*, 1456–1465.

(18) Bryenton, K. R.; Adeleke, A. A.; Dale, S. G.; Johnson, E. R. Delocalization error: The greatest outstanding challenge in density-functional theory. *WIREs Computational Molecular Science* **2023**, *13*, e1631.
